# Control and Eradication Programs for Six Cattle Diseases in the Netherlands

**DOI:** 10.3389/fvets.2021.670419

**Published:** 2021-08-18

**Authors:** I. M. G. A. Santman-Berends, M. H. Mars, M. F. Weber, L. van Duijn, H. W. F. Waldeck, M. M. Biesheuvel, K. M. J. A. van den Brink, T. Dijkstra, J. J. Hodnik, S. A. J. Strain, A. de Roo, A. M. B. Veldhuis, G. van Schaik

**Affiliations:** ^1^Department of Research and Development, Royal GD, Deventer, Netherlands; ^2^Department of Population Health Sciences, Faculty of Veterinary Medicine, Utrecht University, Utrecht, Netherlands; ^3^Department of Cattle Health, Royal GD, Deventer, Netherlands; ^4^Department of Production Animal Health, Faculty of Veterinary Medicine, University of Calgary, Calgary, AB, Canada; ^5^Veterinary Faculty, University of Ljubljana, Ljubljana, Slovenia; ^6^Animal Health and Welfare Northern Ireland, Dungannon, United Kingdom

**Keywords:** disease control, sound control, endemic diseases, control programs, monitoring, surveillance, dairy, beef

## Abstract

Within the European Union, infectious cattle diseases are categorized in the Animal Health Law. No strict EU regulations exist for control, evidence of disease freedom, and surveillance of diseases listed other than categories A and B. Consequently, EU member states follow their own varying strategies for disease control. The aim of this study was to provide an overview of the control and eradication programs (CPs) for six cattle diseases in the Netherlands between 2009 and 2019 and to highlight characteristics specific to the Dutch situation. All of these diseases were listed as C,D or E in the New Animal Health Law. In the Netherlands, CPs are in place for six endemic cattle diseases: bovine viral diarrhea, infectious bovine rhinotracheitis, salmonellosis, paratuberculosis, leptospirosis, and neosporosis. These CPs have been tailored to the specific situation in the Netherlands: a country with a high cattle density, a high rate of animal movements, a strong dependence on export of dairy products, and a high-quality data-infrastructure. The latter specifically applies to the dairy sector, which is the leading cattle sector in the Netherlands. When a herd enters a CP, generally the within-herd prevalence of infection is estimated in an initial assessment. The outcome creates awareness of the infection status of a herd and also provides an indication of the costs and time to achieve the preferred herd status. Subsequently, the herd enrolls in the control phase of the CP to, if present, eliminate the infection from a herd and a surveillance phase to substantiate the free or low prevalence status over time. The high-quality data infrastructure that results in complete and centrally registered census data on cattle movements provides the opportunity to design CPs while minimizing administrative efforts for the farmer. In the CPs, mostly routinely collected samples are used for surveillance. Where possible, requests for proof of the herd status are sent automatically. Automated detection of risk factors for introduction of new animals originating from a herd without the preferred herd status i.e., free or unsuspected, is in place using centrally registered data. The presented overview may inspire countries that want to develop cost-effective CPs for endemic diseases that are not (yet) regulated at EU level.

## Introduction

As opposed to notifiable cattle diseases listed as category A or B, for cattle diseases listed in a lower categorization (C, D, or E) such as bovine viral diarrhea (BVD), paratuberculosis, infectious bovine rhinotracheitis (IBR), and salmonellosis, no or limited EU regulation exists (referred to as non-EU regulated diseases in the rest of this manuscript) (Regulation (EU) 2016/429). These diseases are often endemic and result in substantial adverse cattle health. Additionally, presence of the diseases results in reduced cattle welfare and increased labor and costs for the farmer (1, 2). Moreover, concerns about the zoonotic potential of *Leptospira* spp. (3), and *Salmonella* spp. (4) have been a major driver to control the infections. Thus, several European countries have implemented national or regional surveillance, control, or eradication programs (5–8).

In this manuscript, the term “Control Programs” (CPs) is applied to programs that may lead to a free or unsuspected (“low-risk”) status from a particular infection at herd level.

Because these programs bring tangible benefits to participating farmers and the dairy processing industry, development of and participation in CPs are strongly supported by farmer organizations, dairy processors and the meat industry (9, 10). The differences between programs in the various EU member states also create difficulties for intra-community trade, as trade may introduce infectious agents into regions or herds where disease freedom has been achieved. The difficulties relate to differences in infection statuses between countries, differently designed disease CPs, and the lack of agreed methodologies to assess and compare confidence of freedom from infection in cattle that are being moved between countries and regions. Although for non-regulated infections no or limited regulations exist at European level, an understanding of equivalence with respect to confidence in freedom from infection is important when seeking to facilitate interstate animal movements, whilst also managing the risk of infection.

In 2018, a COST Action (European Cooperation in Science and Technology) named SOUND-control was initiated that stimulated development of methods that enable the comparison of the output of heterogeneously designed CPs between countries (www.sound-control.eu). As part of this COST Action each of the 32 participating countries, including the Netherlands, provided a comprehensive overview of the CPs in place for non-regulated cattle diseases in their country. This information will form the basis and guide the needs for an eventually developed method to compare outputs of CPs in an objective and uniform manner.

The aim of this paper is to describe the Dutch CPs for six cattle infections i.e., bovine viral diarrhea virus (BVDV), bovine herpes virus type 1 (BoHV-1), *Salmonella enterica* subsp. *enterica* serogroup B and D (*Salmonella*), *Mycobacterium avium* subsp. *paratuberculosis* (*Map*), *Leptospira* serovar Hardjo (*L*. Hardjo) and *Neospora* subsp. *caninum* (*Neospora*) between 2009 and 2019.

## Materials and Methods

### Cattle Population and Definitions

For this study, all Dutch cattle herds that participate in the Dutch Cattle Health Surveillance System (CHSS) (11) were included, at present >98% of all cattle herds. Data on aspects such as milk delivery and animal movements enabled us to assign a cattle herd type to each individual herd:

1. Dairy herds: herds that deliver milk to dairy plants in the Netherlands (Qlip laboratories, Zutphen, the Netherlands).
2. Suckler herds: These herds do not deliver milk and have more than 20 cattle. The majority of animals (>80%) are cows and annually at least one calf is born in these herds (Identification and registration (I&R) data, Netherlands enterprise agency Nederland (RVO), Assen, the Netherlands).
3. Young stock rearing herds: the majority of the cattle (>95%) are female and younger than 2 years of age. The cattle enter the herd at a young age (<3 months) and leave the herd before first calving to a dairy herd (I&R data, RVO).
4. Beef herds: are defined as herds with calves (veal) or older cattle (beef) that, in general, are exclusively moved off-farm to go to slaughter. The majority (>80%) of the cattle are male and in general no calves are born in these herds, and no milk is delivered (I&R data, RVO).
5. Small scale holdings: herds that do not deliver milk to dairy plants and with <20 cattle in total (I&R data, RVO).
6. Other herds: herds that do not fit into the above mentioned criteria (<5% of all cattle herds). This group includes herds with at least 20 head of cattle, that do not deliver milk, that have no births and that do not meet the criteria for beef or veal. This group mainly includes trading farms, herds that just started or almost stopped and other small groups of herds (e.g., petting zoos).

Some CPs involve surveillance on bulk milk samples, which is obviously only applicable to dairy herds. Other surveillance strategies are based on individual test results and can accommodate both dairy and non-dairy herds.

### Description of Control Programs for Cattle Diseases With No or Limited Regulation on EU Level That Are in Place in the Netherlands

Each of the CPs have been tailored to the specific situation in the Netherlands, i.e., a country with high risk of introduction and transmission of infections but also with a high data quality at national level. This enables the use of standardized and targeted sampling in CPs, such as bulk milk sampling (dairy) and slaughterhouse sampling (non-dairy). Additionally, this also enables use of routinely collected data for risk-based surveillance and to support the coordination of the CPs. All six cattle diseases with no or limited regulation on EU level for which CPs are in place are endemic in the Netherlands, at varying prevalence's of infection (see Results section).

In general, three phases are distinguished when conducting a CP, (i) initial assessment in which the (apparent or true) prevalence of infection in the herd is estimated as the starting point for disease control, (ii) control phase in which actions are initiated to eliminate the infection if present or to reduce the prevalence if eradication is impossible and (iii) the surveillance phase that monitors the achieved preferred disease status (free or unsuspected depending on the disease) and take action when (re-)introduction of the infection is detected. Within the CPs, herds are assigned with one of six different disease statuses that are defined as follows:

- Free: is achieved after whole herd screening without evidence of infection (period differs depending on the disease) or after a prolonged period of proof of an unsuspected status. More information on the definition of the free status is described in sections BVDV to Leptospirosis.
- Unsuspected: screening of a sample of animals in the herd (e.g., bulk milk, sample of young stock or random sample of cattle), yields no indication of infection.
- Suspended: evidence or action is needed to prove that the herd is still free or unsuspected. The herd is within the time frame that is set to deliver the requested evidence. The herd needs to test cattle to prove that they are free of infection (after introduction of cattle originating from herds that are not classified as free or after lacking to provide evidence of freedom within the standard terms set out in the CPs), or have to prove that the herd is free of infection again after reintroduction of the disease (and removal of the infected animals).
- Unknown: the herd is a still participant in the CP but evidence or action is needed to prove that the herd is still free or unsuspected. The herd is outside the time frame that is set to deliver the requested evidence.
- Infected: Presence of infection has been established.
- Controlled: actions are taken to eliminate the infection with the aim to achieve the free or unsuspected status. These actions test and cull, vaccination, or treatment and monitoring the subsequent status. An example of this status “controlled” is the “vaccinated” status for BoHV-1.

For BVDV, BoHV-1 and *L*. Hardjo the highest herd status that can be achieved is the free status, although for BVDV and BoHV-1 herds can also obtain an unsuspected status. For *Salmonella* spp. the highest preferred health status that can be achieved in the Netherlands is “unsuspected.” For *Map*, the preferred herd status is status A and status 10, which are equivalent to an unsuspected (status A) or free status (status 10). For *Neospora*, herds cannot achieve a free or unsuspected status and the only stages that are recognized are “participating” or “not participating” in the monitoring program. Each CP has its own aim and design which is outlined below in more detail. Additional links to detailed regulatory CP information for each included infection are provided in Appendix 1.

#### BVDV

Bovine viral diarrhea virus (BVDV) is a member of the pestiviruses and the causal agent of bovine viral diarrhea (BVD). The virus can be transmitted both horizontally, leading to transiently infected cattle (TI), and vertically. In transiently infected cows, infections with BVDV may sometimes lead to severe clinical signs (12, 13). Vertical transmission in the first trimester of gestation can result in a Trojan cow (TR) that carries a persistently infected calf (PI) (14). These PI cattle are the most important source of virus transmission because they constantly shed large amounts of virus (15).

In the Netherlands, up to 2018, a voluntary BVDV CP was in place in which dairy and non-dairy herds could participate. Since 2018, the CP was implemented on a national level while its design was slightly modified. Since then, the Dutch dairy sector (ZuivelNL, The Hague, The Netherlands) has required dairy farmers to participate in this national program (at their own expense). The aim of the national BVDV program is to eliminate BVDV from dairy herds and prevent reintroduction of the virus. For non-dairy herds, up to now, participation remains voluntary. The beef and veal producing industry have committed themselves to participate in the BVDV eradication in the coming years. It is anticipated that non-dairy herds will seek to control and eliminate BVDV and hopes are that the Netherlands will eventually become BVDV free.

**Figure 1:**
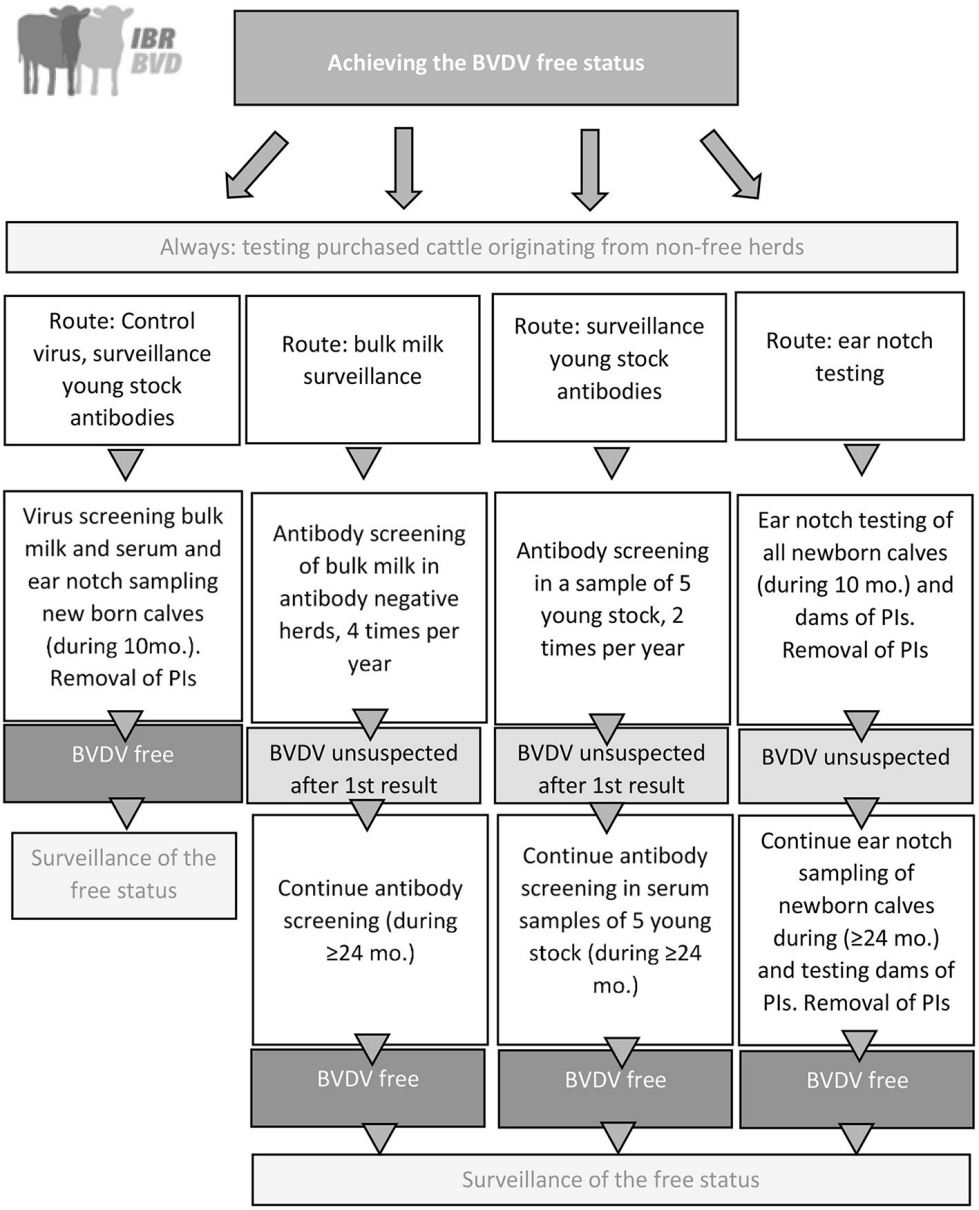
Graphical overview of the national BVDV control program in the Netherlands that was implemented in 2018. PI, Persistently infected animal.

In the BVDV program, dairy farmers choose one of four different routes to achieve the BVDV free status (Figure 1). These routes differ in the duration to obtain the free status, but also in costs and labor involved. Each route aims to detect BVDV either directly by testing for virus or indirectly by testing for antibodies against BVDV (16, 17). To support farmers in their choice for a route it is advised (but not mandatory) to evaluate the herd status by testing bulk milk and serum samples of five young stock aged 8–12 months prior to enrolment in the CP. The first route consists of two phases a control phase followed by a surveillance phase. In the control phase all cattle in the herd are screened for virus (utilizing serum of all non-lactating animals and bulk milk followed by serum testing of all individual lactating cattle in case of a positive bulk milk test result). If persistently infected (PI) animals are detected, it is mandatory to remove them. All calves born in the subsequent 10 months are screened for the presence of BVDV by testing ear notch samples or serum samples collected at >30 days of age. After ten months of negative test results in newborn calves, the BVDV free status is assigned. In the surveillance phase, the free status is monitored twice a year by testing for antibodies in five young stock between eight and twelve months old. Vaccinating herds are recommended to select cattle that are not (yet) vaccinated for the biennial antibody evaluation to prevent interference of vaccination with the test results. In the other three routes, the BVDV status is monitored by testing for antibodies in bulk milk, antibodies in serum samples of young stock, or testing for virus in ear notches. After 24–36 months (depending on the route) without any indication of BVDV presence in the herd, the BVDV-free status is achieved. When antibodies are detected in the bulk milk or young stock route, herds are redirected to the route “control virus and monitoring antibodies in young stock.” Non-dairy herds that want to be classified as BVDV free can follow the route “control virus and monitoring antibodies in young stock” or the route “ear notch testing.”

In all routes the risk of purchase of cattle from herds without a BVDV free status is monitored. More information on this can be found in section Management and coordination of disease control programs.

#### BoHV-1

Bovine herpes virus type 1 (BoHV-1) is the causal agent of infectious bovine rhinotracheitis (IBR), infectious pustular vulvovaginitis (IPV) and infectious pustular balanoposthitis (IPB). Infection with BoHV-1 in cattle can occur sub clinically but can also lead to severe respiratory symptoms and abortion (18). Seropositive cattle remain latently infected throughout their lives and stress can induce virus reactivation and intermittent excretion of the virus, resulting in continuous risk of spread to susceptible cattle (19, 20). Purchase of cattle and direct contacts between cattle from different herds are the major risk factors for reintroduction of BoHV-1 (21).

Since 2018, a national BoHV-1 CP was implemented in the Netherlands after more than 17 years of having a voluntary CP (16). Since then, the dairy processing industry (ZuivelNL, the Hague, the Netherlands) have required all Dutch dairy herds to control BoHV-1 in their herds. For non-dairy herds, participation in the CP remains voluntary. Participating farmers pay for the costs of the CP. The aim of the national BoHV-1 CP is to control and subsequently eliminate BoHV-1 at herd level and to eventually achieve a BoHV-1 free dairy cattle sector.

Prior to enrollment of participation in the BoHV-1 CP, it is advised to start with a herd screening by conducting a BoHV-1 gE-antibody test in bulk milk (in dairy herds) or individual serological screening for antibodies in a sample of at least three cattle (the oldest ones) in non-dairy herds. In dairy herds, this initial screening can result in two outcomes: more than 10% of the cattle is gE-antibody positive, or at most 10% of the cattle is antibody positive. In non-dairy herds this initial screening only provides a rough indication whether BoHV-1 is present in the herd and whether it is best to enroll in the CP or to start vaccinating. Sampling the oldest three cattle in non-dairy herds is based on the observations that BoHV-1 has a high transmission value in a susceptible herd, and a new introduction will generally result in a major outbreak with at least 70% antibody positive cows (22, 23). A sample of three animals should be sufficient to detect this level of transmission in the herd. Additionally, infected cattle remain antibody positive throughout their lives. When the oldest cows test antibody negative, it is likely that the virus has not spread for a substantial period.

**Figure 2:**
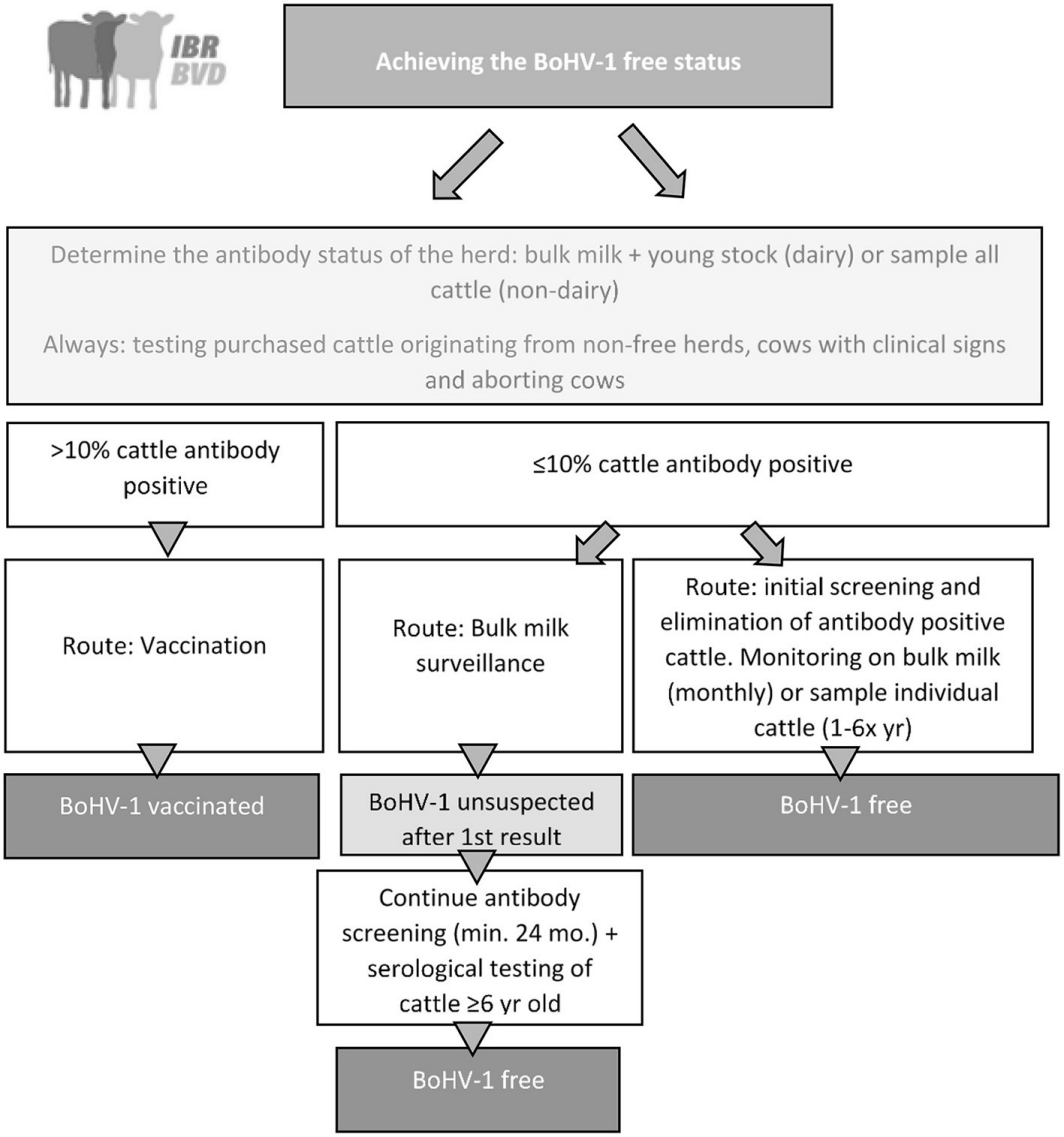
Graphical overview of the national BoHV-1 control program in the Netherlands that was implemented in 2018.

When there is indication that more than 10% of the cattle is gE-antibody positive, removal of all antibody positive cattle will often not be feasible given the high costs involved, and the herd is advised to move to the vaccination route. In this route, the veterinarian vaccinates all cattle in the herd ≥3 months old twice a year with a gE-negative marker vaccine. In the Netherlands, only gE-negative marker vaccines are allowed to be used for BoHV-1. A declaration of vaccination is sent by the veterinarian to the CP's coordinator (Royal GD), and the vaccinated status is assigned. Control of the infection by vaccination will prevent major BoHV-1 outbreaks in the herd (24) and (25). Subsequently, over time the gE-positive cattle will be culled and replaced by gE-seronegative young stock, which will result in a slow disappearance of cows with gE-antibodies and thus in a reduction of gE-antibodies in bulk milk. Therefore, annual screening of bulk milk for antibodies is advised. When there is an indication that the gE-antibody level has decreased to at most 10%, the farmer can opt to change to the route in which a BoHV-1 unsuspected or BoHV-1 free status can be achieved (Figure 2). When <10% of the cattle is gE-antibody positive, two routes can be followed to achieve a BoHV-1 free status.

The first route “bulk milk route,” involves bulk milk monitoring, which takes at least 2 years. In this bulk milk route, bulk milk is screened for antibodies against BoHV1 gE. When no antibodies are found, the herd receives the status BoHV-1 unsuspected and enters the surveillance phase in which the bulk milk is screened on a monthly interval for the presence of antibodies. After 24 months of antibody negative results, the herd can opt to be classified BoHV-1 free. To obtain this status, all cattle of 6 years and older as well as cattle that were introduced in the herd after the initial assessment of the CP have to be individually screened for BoHV-1 antibodies. If no gE-antibodies are found the herd receives a BoHV-1 free status that will again be monitored by monthly bulk milk antibody testing (Figure 2). Any antibody positive cattle have to be removed from the herd, and thereafter the BoHV-1 free status must be substantiated by testing bulk milk in dairy herds 4–8 weeks later. The second route “Initial screening, elimination of infection and monitoring through bulk milk,” is costlier but faster. When a herd (dairy or non-dairy) starts to participate in the route in which herds are fully screened to receive a BoHV-1 free status within a short period, all cattle ≥12 months old are serologically screened for antibodies against BoHV-1. When calves (<12 months old) originating from a herd without a BoHV-1 free status are present, all cattle >7 days old have to be serologically screened. Any antibody positive cattle will be removed from the herd. If a subsequent sample of cattle 4–8 weeks later yields negative test results, the BoHV-1 free status is assigned. This status will be monitored by monthly testing in bulk milk in dairy herds or by slaughterhouse surveillance in non-dairy herds where, depending on the herd size and frequency of sending cattle to slaughter, one to six cattle are selected for BoHV-1 antibodies at slaughter per year (Figure 2).

In the CP, the risk of purchase of cattle from herds without a BoHV-1 free status is monitored as are cattle that show clinical signs that may be indicative of a BoHV-1 infection, such as respiratory symptoms or abortion. In the CP these cattle have to be tested for presence of BoHV-1 antibodies or virus in case of respiratory symptoms and if present subsequent actions need to be taken. Further details can be found in section Management and coordination of disease control programs.

#### Leptospirosis

Leptospirosis in cattle is a zoonotic infection that is predominantly caused by *Leptospira interrogans* serovar Hardjo type prajitno and *Leptospira borgpetersenii* serovar Hardjo type bovis (26). In the Netherlands, serovar Hardjo type bovis has been described in both cattle (27–30) and cattle farmers (31) and is referred to as *L*. Hardjo in the remainder of this paper. Generally, *L*. Hardjo enters the body through the mucous membrane of eyes, nose, uterus, or mouth. Transmission of the bacteria mainly occurs through urine or with urine contaminated feed or water from infected cattle (32). Once infected, animals often become carriers that intermittently excrete the bacteria into the environment and therefore are a source of infection for other animals (33). Infection of *L*. Hardjo in cattle may evolve without any clinical signs but can also lead to loss of milk production, abortions and reproductive problems (34, 35). In the Netherlands, currently, no vaccines are registered for *L*. Hardjo and vaccination is therefore not part of control of the infection.

**Figure 3:**
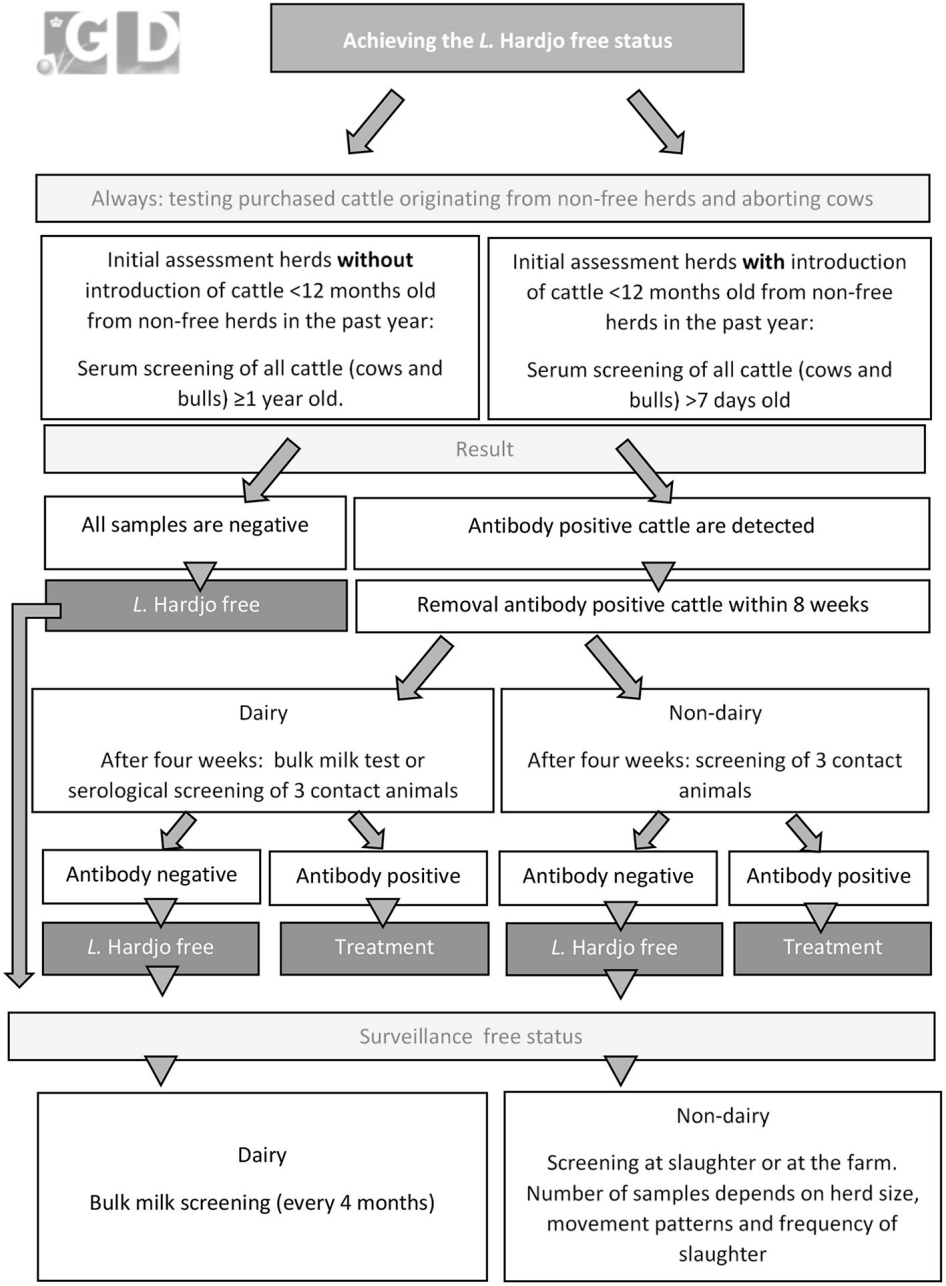
Graphical overview of the *L*. Hardjo-control program in the Netherlands that was implemented in 1994.

Because *L*. Hardjo caused clinical disease in farmers in the Netherlands in the nineties, a CP was developed in 1994. Since 2005, the Dutch dairy sector demands a *L*. Hardjo free status for dairy herds delivering milk in the Netherlands. For non-dairy herds, participation in the CP is voluntary. A graphical overview of the CP is presented in Figure 3. At enrolment in the CP, all cattle ≥12 months old in the herd are tested for antibodies against *L*. Hardjo (36). In herds with introduction of cattle from non-free herds in the previous year, the calves >7 days old are also tested. If no antibodies are detected, the herd is classified as *L*. Hardjo free. When antibodies are detected, the antibody positive animals must be removed. Four to eight weeks later, either a bulk milk sample or serological samples of young stock are tested (depending on the age of the removed cattle), to check the *L*. Hardjo status of the dairy herd. When antibody positive cattle are detected during this second evaluation, there is confirmation that there is an active *L*. Hardjo infection in the herd. In non-dairy herds a sample of contact animals is tested 4–8 weeks after removal. When active circulation with *L*. Hardjo is detected, treatment of all cattle in the herd with dihydrostreptomycin (25 mg/kg I.M.) is advised. After treatment, the herd status is changed into “controlled” and the dairy processors determine how long the farm can deliver milk under this status. To survey for transmission in treated herds, every 6 months a seronegative sentinel group of animals ≥2 years old are serologically examined for *L*. Hardjo antibodies.

After the *L*. Hardjo free status is assigned to a dairy herd this status is monitored every 4 months through antibody evaluation of bulk milk. In non-dairy The *L*. Hardjo free status of non-dairy herds is monitored through antibody testing of sera collected at slaughter. The frequency of testing at slaughter varies between one and six cattle per year, depending on herd size, on- and off-farm movement patterns, and slaughter frequency.

The risk of introductions of cattle from herds without a *L*. Hardjo free status is controlled by serology testing of introduced cattle. Additionally, farmers are obliged to submit samples of aborting cattle to evaluate the presence of *L*. Hardjo. More information on the logistics involved in controlling these risk factors can be found in section Management and coordination of disease control programs.

#### Salmonellosis

*Salmonella enterica* subsp. *enterica* infections (*Salmonella* spp.) are of concern to the cattle industry as cause of economic and welfare losses in infected herds and as risk of zoonotic infection (37, 38). The most prevalent serogroups in Dutch dairy cattle are serogroups B (including serovar Typhimurium) and D (including serovar Dublin) (39). The common route of transmission between cattle is fecal-oral infection, and consequently contamination of the environment, feed and water play an important role in the epidemiology (40). Due to differences in herd management, a large variation was observed in the rate of transmission within herds (41). Introduction of cattle or slurry from other herds are important routes of transmission of the infection between herds (37, 42–44). Both herd management and culling of persistently infected *Salmonella* spp. carriers play an important role in the control of the infection in infected herds (40, 45).

A voluntary CP for *Salmonella* spp. in Dutch cattle herds (both dairy and non-dairy herds) was initiated in 2000 by Royal GD to enable low-risk trade of cattle between herds, to alert farmers to a *Salmonella* spp. infection in their herd, and to reduce human exposure to *Salmonella* spp. (43). Testing sera and bulk milk samples by ELISA for antibodies against *Salmonella* spp. serogroups B and D plays an important role in the initial assessment and surveillance phases of the programme. In 2020, the CP was slightly modified and the initial assessment in dairy herds now consists of testing bulk milk samples at 4-month intervals. The initial assessment in non-dairy herds consists of testing sera of the 10 youngest cattle over 90 days of age that have been present in the herd for at least 70 days. With this number, the negative predicted value was estimated at 94% (95% CI: 91–96%, unpublished data), when a design within-herd prevalence of 0.1, a diagnostic Se of 94.4% (95% CI: 72.7–99.9) (46) and a test Sp of 99.3% (95% CI: 97.7–99.7) (47) were used. The threshold of 90 days old is used to avoid interference of maternal antibodies and cattle have to be present in the herd for at least 70 days to ensure that the test result is indicative for the *Salmonella* spp. status of the current herd. Test-negative herds are assigned the status *Salmonella* spp. unsuspected. Surveillance of unsuspected herds consists of testing bulk milk samples at 4-month intervals (dairy herds) and twice a year testing of sera of the 5 youngest cattle over 90 days of age that have been present in the herd for at least 70 days (non-dairy herds). Additionally, risk based surveillance is applied by surveillance for any positive test results of samples submitted from the herd from potential clinical cases (including serology of any aborting cattle and bacterial cultures from post mortem samples and feces) and serological testing of any cattle introduced from herds without an unsuspected status. Positive test results in any of the routes result in suspension of the unsuspected herd status until follow up testing shows that the infection is no longer spreading. Infected herds are advised to control the infection by preventive management measures and identification and culling of *Salmonella* spp. carriers (45).

**Figure 4:**
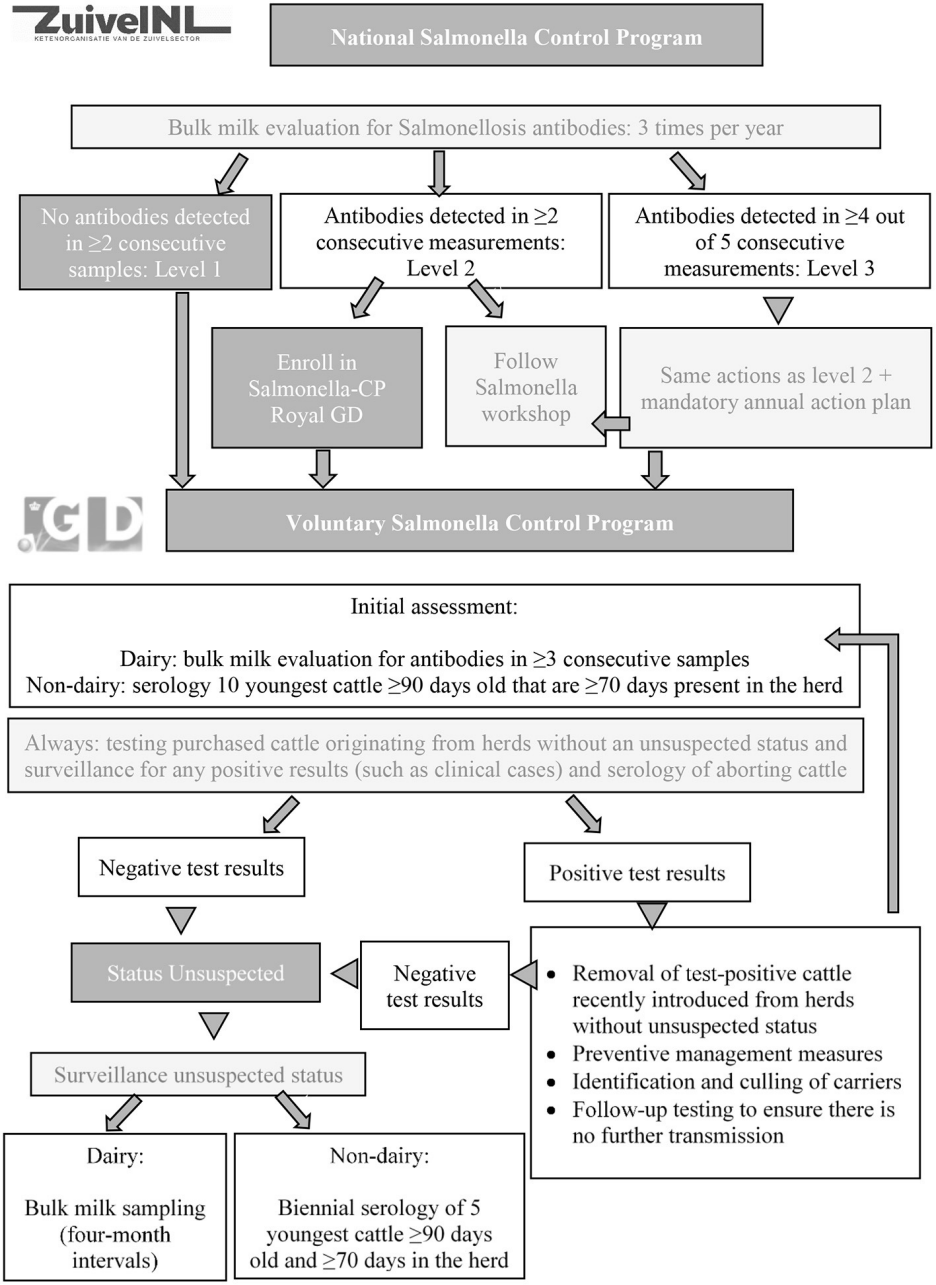
Graphical overview of the national *Salmonella* spp. control program (ZuivelNL, the Hague, the Netherlands) and the voluntary *Salmonella* spp. unsuspected control program (Royal GD, Deventer, the Netherlands) in the Netherlands that was implemented in 2009.

In 2009, the Dutch dairy processing industry (ZuivelNL, The Hague, The Netherlands) implemented a mandatory CP in addition to the pre-existing voluntary CP. The aim of the mandatory CP is to reduce the *Salmonella* spp. prevalence in the dairy processing industry (Figure 4). Like the voluntary CP, this mandatory CP involves bulk milk screening for antibodies focused on detection of *Salmonella* spp. serogroups B and D at 4-month intervals. Based on the bulk milk results (antibodies detected or not detected), herds are classified in one of three categories. Consistently bulk milk antibody negative herds are classified as Level 1. Herds with at least two subsequent antibody positive bulk milk results are classified as Level 2. Herds in which antibodies are detected in at least four out of the five most recent bulk milk evaluations are classified as Level 3. Level 2 and 3 herds are obliged by the terms of delivery of their dairy processor to take control efforts. These efforts range from either a risk assessment or participation in the voluntary CP of Royal GD at Level 2, to an annual mandatory action plan including both preventive management measures and identification and culling of active *Salmonella* spp. carriers at Level 3. Herds that are assigned Level 3 for more than 3 years are obliged to seek advice of one of five specifically trained veterinarians during a herd visit before drawing their next action plan. At this stage, the dairy processors closely monitor the efforts of the farmer, to ensure that the drawn action plan is followed through.

#### Paratuberculosis

Paratuberculosis (or Johne's disease) in cattle is an infectious disease caused by Mycobacterium avium subsp. paratuberculosis (*Map*). The disease is widespread world-wide and causes significant economic losses (48–50). The infection in cattle is chronic and slowly progressive and often remains restricted to the intestinal tract. Clinical signs include diarrhea, weight loss, reduced milk production and fertility and eventually mortality (6).

Concerns about the zoonotic potential of *Map* are the major driver to control *Map* in cattle populations worldwide. In 1997, Royal GD developed a plan to eradicate paratuberculosis in the Netherlands (51). This resulted in the initiation of the voluntary Intensive Paratuberculosis Programme (IPP) aiming to eliminate the infection from known infected herds, reduce between-herd transmission and enabling low-risk trade of cattle between herds (51–53). In addition to this CP, in 2006, a Milk Quality Assurance Programme (MQAP) (54) was started on a voluntary basis which became mandatory for Dutch dairy herds from 2010 on (55). The aim of this MQAP is to reduce the concentration of *Map* in milk delivered to the milk processors.

**Figure 5:**
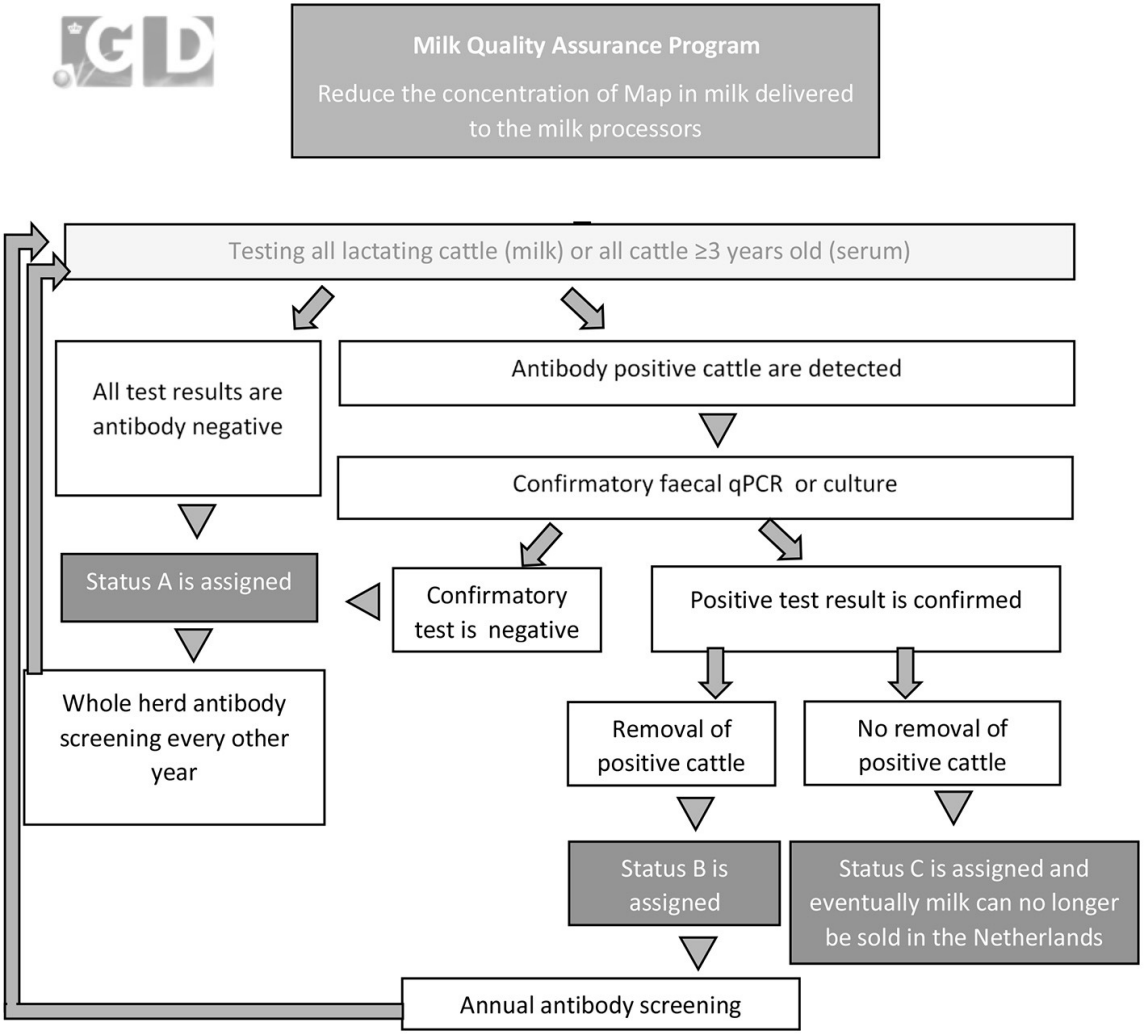
Graphical overview of the mandatory Milk Quality Assurance Program for paratuberculosis (Royal GD, Deventer, the Netherlands) in the Netherlands that was initiated in 2006.

In the MQAP, herds are assigned a status based on herd examinations consisting of individual testing of either all lactating cattle or all cattle over 3 years of age for presence of antibodies against *Map* (Figure 5). If all individuals are test negative, status A status is assigned (low risk herd). If antibody positive cattle are detected farmers can opt to confirm these results by fecal PCR-assay or culture. If all positive cattle are removed from the herd, status B is assigned. If any positive cattle are retained, the herd is assigned status C. Herds with status C, are eventually no longer allowed to deliver milk to dairy processors in the Netherlands. Herd examinations are done annually (status B and C) or biennially (status A). Herds with status A can introduce cattle from other herds with status A or an unsuspected status in the IPP without restrictions. Adult cattle introduced from herds with a lower or unknown status must pass a serum-ELISA test with a negative result (56).

As an alternative to participation in MQAP, farmers can participate in the IPP. The IPP describes 6 classifications for herds, with increasing confidence of freedom from infection (51–53, 57). The IPP distinguishes an initial assessment (status 5–9) and a surveillance phase (status 10, also known as “*Map* free”). At enrolment the herd is assigned status 5 and all cattle over 3 years of age are tested by serum antibody ELISA followed confirmatory fecal culture or PCR assay. If the screening is negative, the herd progresses to status 6. Subsequent annual herd examinations consist of culture or qPCR of pooled fecal samples of all cattle over 2 years of age (58). Annual progression from status 6–10 occurs with each negative herd examination. Surveillance of status 10 herds is done by biennial herd examinations. Any positive test result means loss of the herd status. Herds in IPP can only purchase cattle from herds with equal or higher certification status. If cattle are purchased from a herd with a lower status, the herd status is reduced. More detailed information on the MQAP and IPP is provided in Whittington et al. (6) and Geraghty et al. (59). Given that only a small proportion of herds participate in the IPP (<2% of the herds at present), we will focus on the MQAP in the remainder of this paper.

#### Neosporosis

*Neospora* subsp. *caninum* (*Neospora*) is an apicomplexan protozoon, an important cause of abortion in cattle worldwide (60). Horizontal transmission of *Neospora* in cattle occurs through ingestion of feed contaminated with fecal oocysts shed by infected dogs and in dogs through the ingestion of infected bovine placentae (61, 62). However, the main route of transmission in cattle is vertically from cow to calf during gestation from congenitally infected cows transmitting the infection to their offspring (63). Infections with *Neospora* are known to be associated with abortion storms which can result in significant losses for farmers.

**Figure 6:**
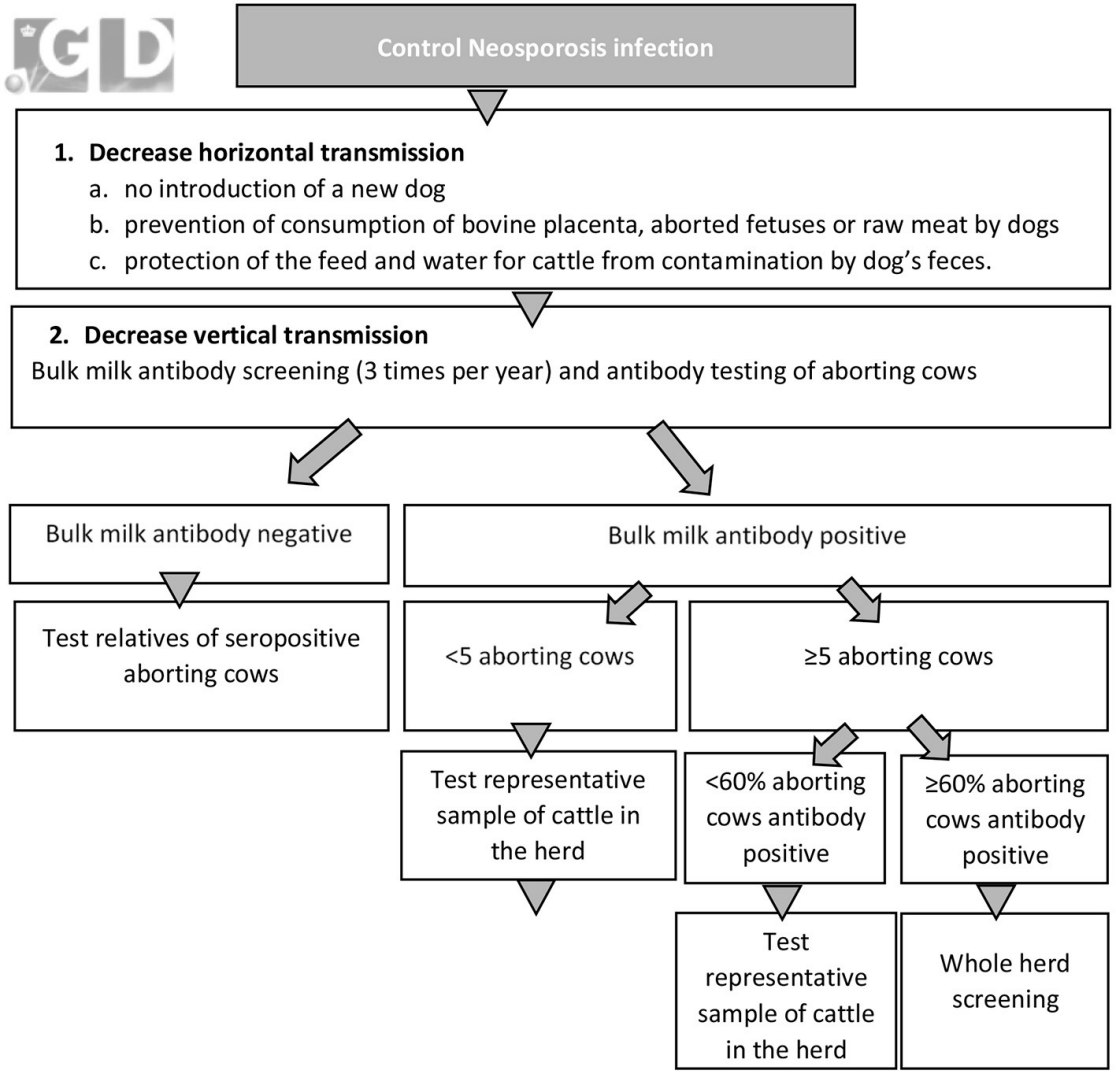
Graphical overview of the voluntary neosporosis control program (Royal GD, Deventer, the Netherlands) in the Netherlands that was implemented in 2003.

In 2003, Royal GD developed a voluntary *Neospora* CP for dairy herds, aiming to control neosporosis and to reduce the associated reproductive losses. The CP consists of routinely antibody screening of bulk milk at 4-month intervals and serological antibody screening of aborting cattle. When antibodies are detected a follow-up screening is conducted (Figure 6). The aim of the follow-up screening is to get an overview of the transmission route i.e., age-clustering and the serological status of a family line, the within-herd seroprevalence, and the time frame in which post-natal infection may influence the infection status of individual animals. Based on these more detailed results of the within-herd status the farmer and his/her veterinarian develop a tailored plan to control *Neospora* in the herd (Figure 6). Specific aspects that should be considered in the control of *Neospora* beside specific dog management practices include culling of seropositive (aborting) cows, culling of heifer calves born from seropositive dams, testing of purchased cattle, use of semen from beef bulls for seropositive cows and sexed semen on seronegative cows. In case of genetically valuable seropositive cows it is advised to apply embryo transfer and use seronegative donor animals.

### Management and Coordination of Disease Control Programs

In the Netherlands, a good quality infrastructure is present for collecting bulk milk samples (quality control for milk), individual milk samples (collected for milk production registration) and routine collection of data. These samples and data can automatically be used in support of the CP with informed consent of the farmer, which is obtained at enrolment in the CPs. Using routinely collected samples and data, the CPs carried out in the Netherlands are labor sparing and cost-efficient. All programs are managed by one organization (GD) with a large commercial veterinary laboratory. This lab is accredited by the relevant authorities, as well as all diagnostic tests used for the CPs. In some cases, diagnostic results from other labs are also allowed in the CPs. These labs are on a list and accredited by the national reference institute (WBVR). The labs can use their own in-house or commercial kits and WBVR assures with proficiency tests that diagnostic test validity is comparable between the labs.

Data is routinely collected in an objective and standardized way on a national level, enabling optimization, and automatization of processes within the CPs. For coordination of all control efforts, cattle movement data from the identification and registration system (I&R database, RVO, Assen, the Netherlands) is combined within automated Certification Coordination software Programs (CCP) that evaluate whether the herd meets all criteria set by the CP. When results are needed for all or a sample of cattle, the CCP automatically consults the I&R database to determine for which cattle at what moment a test result is needed and both farmer and veterinarian are notified accordingly. Notification is done by regular mail and through email. When the subsequent samples are submitted and the laboratory test results become available, the CCP automatically processes the test results in the CP, adapts the status if needed and informs farmers and veterinarians of the result and the CP status by either mail or email.

In the BVDV, BoHV-1, *L*. Hardjo, *Salmonella* spp. (unsuspected CP) and *Map* (IPP as well as MQAP) programs, any introductions of cattle into participating herds are identified real-time using data from the national I&R database, in which all cattle movements are recorded. This information is processed by the CCP software within a day and an observation status is assigned to the herd if the herd of origin had a lower herd status. Subsequently the farmer and veterinarian are notified if removal or testing of the introduced cattle is required.

In CPs for BVDV, BoHV-1, *L*. Hardjo, *Salmonella* spp. and *Neospora* bulk milk samples are tested. These bulk milk samples are routinely collected at the time of on-farm collection of milk by the dairy processors and tested for milk quality purposes at Qlip laboratories (Zutphen, the Netherlands). If a dairy farmer enrolls in a CP in which bulk milk testing is part of the intake, control or surveillance phase, an automated request is sent to Qlip to forward a bulk milk sample to Royal GD for testing. The farmer receives the test result automatically without having to take any action.

For CPs where risk-based testing of aborting cows is included, the CCP detects samples of aborting cattle that are submitted for mandatory brucellosis surveillance. When a herd participate in the CP for BVDV, BoHV-1, *L*. Hardjo, *Salmonella* spp. or *Neospora* samples are automatically screened for presence of these infections.

### Quantifying the Effects of Control Programs

#### Prevalence Surveys

Since 2004, the Dutch cattle industry monitors the prevalence of endemic cattle diseases based on antibody or virus testing. Every 2 years, the cattle industry decides on a number of non-regulated cattle diseases to include in a biennial prevalence survey. The presented survey results represent the apparent prevalence which are referred to as “prevalence” in the remainder of this paper.

Diseases to be included in the survey are selected based on relevance to the industry with regard to costs, impact on animal health and welfare, public health and monitoring progress of control efforts. The selected diseases and herd types for the prevalence surveys also depend on the participation in the CPs i.e., when the participation rate of the CP approaches 100%, a dedicated prevalence survey is not relevant as the data gathered in the CPs provide sufficient information to assess disease prevalence.

For BVDV, BoHV-1, *L*. Hardjo, *Salmonella* spp, and *Map*, herds were screened for the presence of antibodies. Two-stage sampling is applied, and the sample size was determined using WinEpiscope 2.0 (64). For sample size calculation to determine the herd prevalence, an assumption has to be made for the expected herd-level prevalence. If available, the expected prevalence is based on a prevalence estimate from an earlier study. When no former prevalence estimates are available, a 50% herd-level prevalence was assumed, leading to the highest number of herds to be sampled. Additionally, the level of confidence and acceptable error around the herd-level prevalence estimate has to be included in the sample size calculation. In our prevalence surveys the confidence level is set at 95% and the acceptable error at 5%.

For detection of infection within a herd, either bulk milk screening or individual serological screening was applied. In the case of individual screening, an expert opinion-based assumption was made for the expected within-herd prevalence in infected herds to calculate the number of animals to be sampled. For BVDV, BoHV-1, *L*. Hardjo and *Salmonella* spp, a within-herd prevalence of more than 50% in the target population was assumed when an active infection was present, and five random animals from the target group were sampled per herd. For *Map* all cattle ≥3 years old were sampled to enable detection of a low within-herd *Map* prevalence. The herd target population differs depending on the infection and includes the cattle population in which it is most likely to detect an active infection if present (risk-based). For BoHV-1 and *L*. Hardjo, the target population includes cattle ≥2 years old. For BVDV, the target population included calves between 8 and 12 months of age, which are tested for presence of antibodies indicative for BVDV transmission in the herd. For *Salmonella* spp. calves in the age of 3–6 months were included as target population. The sampling process is described in more detail in Veldhuis et al. (65) and Veldhuis et al. (66).

*Neospora* was not included in the prevalence surveys. Therefore, the evaluation of the infection pressure over time was based on post mortem and serological testing of aborting cattle conducted at Royal GD between 2004 and 2019. The percentage of all aborted fetuses submitted for post mortem examination and serum samples of aborting cows in which *Neospora* was diagnosed as the most likely cause for abortion is monitored on a quarterly basis in the CHSS. For this study, the results obtained since 2004 were summarized.

#### Association Between a Favorable Herd Health Status in a CP and Mortality

The CHSS has been in place since 2002 and consists of several surveillance components that combine enhanced passive reporting, diagnostic and post-mortem examinations, random surveys for prevalence estimation of endemic diseases, and quarterly data analysis (11). The aim of the data-analysis component, which is called the Trend Analysis Surveillance Component (TASC), is to monitor trends and developments in cattle health using routine census data. An important parameter in the TASC is cattle mortality. Each quarter of the year, multiple key indicators that describe mortality in cows and several age groups of calves are analyzed using population-averaged Poisson regression models (11). The description of the key indicators, definition, and calculation method of mortality can be found in Santman-Berends et al. (67). Besides the trend in time, the association between mortality and several herd characteristics are evaluated such as herd size, location, purchase, milk production, antimicrobial usage, and herd health status. For dairy herds, the association between the herd health status and mortality were evaluated for four infections (BVDV, BoHV-1, *Salmonella* spp., and *Map*) between 2015 and 2019. For suckler herds, the association between mortality and the herd health status for three infections (BVDV, BoHV-1, and *L*. Hardjo) was assessed in the same period. Given the large sample of included herds (more than 98% of the total number of cattle herds), only associations with a *P* < 0.01 were presented as significant. Interaction terms were not evaluated.

## Results

### Characteristics Dutch Cattle Population

In 2019 there were 34,316 cattle holdings in the Netherlands, of which 45% were dairy herds, 33% are small scale holdings, 9% were suckler herds and 14% were other types of cattle herds (Table 1). The herd size differed significantly between herd types and ranged from, on average, five cattle in small-scale herds to on average 642 calves in veal producing herds (Table 1). Whereas, suckler herds show a seasonal calving pattern, with most calvings occurring in spring time, seasonal calving is generally not observed in Dutch dairy herds. Therefore, a rather constant amount of milk is produced by dairy herds year round.

**Table 1:** Number of cattle herds and average herd size per cattle herd type in the Netherlands in 2019.

**Herd types**	**Number of herds**	**Average herd size (total number of cattle)**	**Herds with introduction of cattle in 2019**
Dairy herds	15,550	157 (103 cattle ≥2 years)	1 or 2 cattle: 9% >2 cattle: 43%
Suckler herds	3,089	60 (30 cattle ≥2 years)	1 or 2 cattle: 19% >2 cattle: 44%
Beef herds	933	100	≈100%
Veal herds	1,776	642	≈100%
Young stock rearing herds	1,799	58	≈100%
Small scale farmers	11,169	5	1 or 2 cattle: 20% >2 cattle: 33%

**Figure 7:**
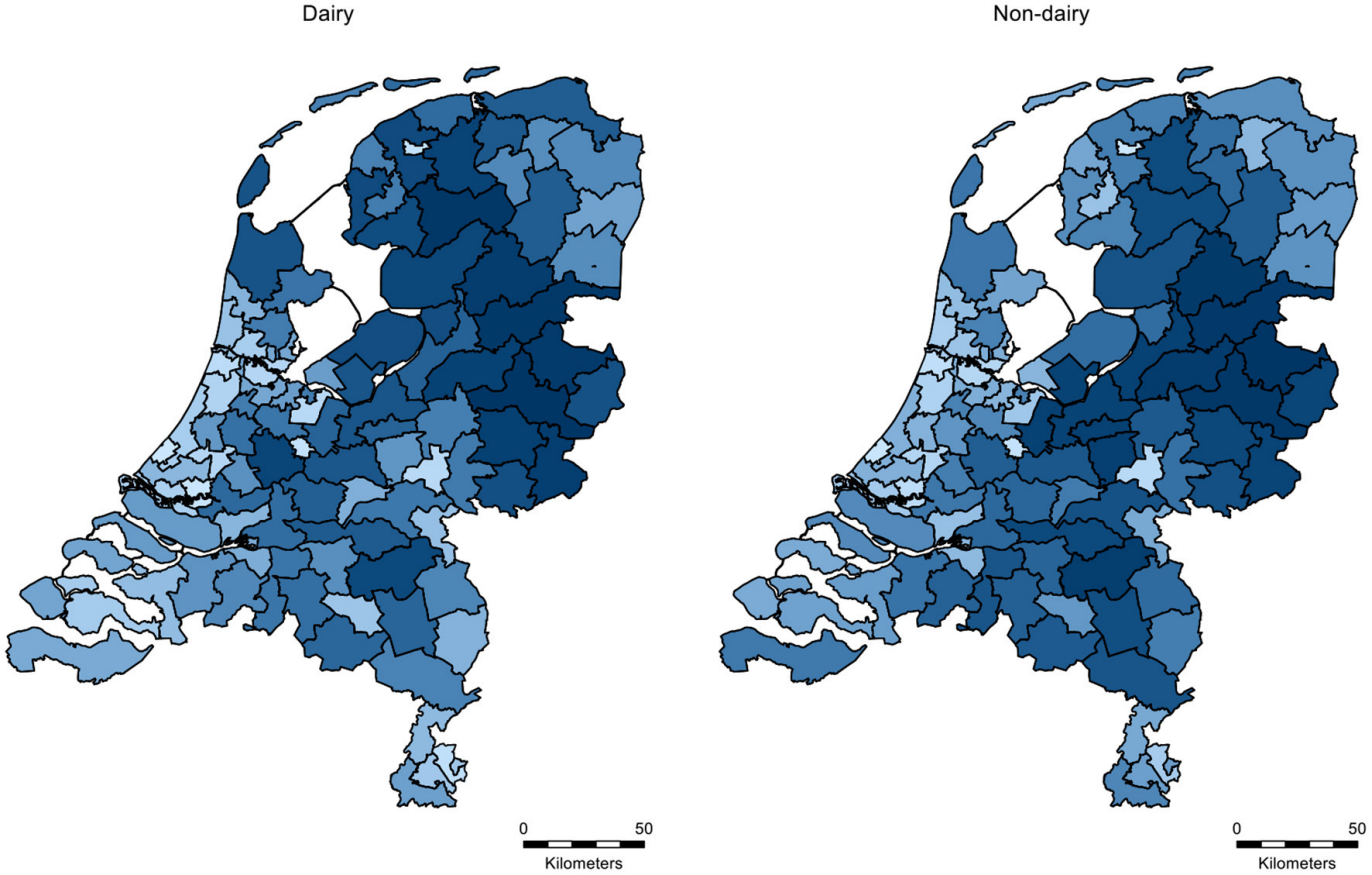
Distribution of the density of dairy and non-dairy herds per 2-digit postal code area in the Netherlands in 2019. Light blue indicates a low herd density and dark blue indicates a high herd density.

The herds are located throughout the country, with the highest densities in the Northern and South-Eastern part of the Netherlands (Figure 7). Overall, the cattle density in the Netherlands (>4 million cattle on 41,526 km^2^ i.e. on average 96 cattle/km^2^, (68), can be classified as high compared to other European countries (68).

There is a high rate of animal movements, both between herds in the Netherlands and with herds in other countries. The Netherlands is one of the countries with the highest number of imported and exported cattle in Europe (69), with more than 750,000 imported and more than 300,000 exported cattle per year. Of the imported cattle, more than 95% are young calves (<1 month of age) imported by the veal producing industry, which are housed indoors and are only moved off-farm to go to another veal producing herd or to slaughter. Animal movements result in a high risk for the introduction and transmission of diseases between herds, and can have a major impact (21, 43, 70, 71).

### Disease Control Programs

#### Participation Rates in Dairy Herds

During the last decade, the participation rates for five out of six CPs increased toward almost 100% in dairy herds, following an obligation to participate in these CPs by the Dutch dairy processing industry. For *L*. Hardjo, farmers delivering milk to any Dutch milk processing plant were obliged to be classified as “free,” which is reflected by 97–98% *L*. Hardjo free dairy herds each year of the studied period (source: CHSS). The remaining herds were mostly in the temporarily observational status because of purchase from a herd without a free status (non-dairy herd or import). In most cases these herds are in fact also free of infection. In the CP's on the other four infections, herds are either classified as having the highest health status i.e., free or unsuspected, or farmers are in the process of obtaining these statuses, hence have to act to achieve the highest health status. Even though the participation rate in dairy herds was close to 100%, the infections are still endemic, and there were still herds in the process of eradicating these infections from their herds.

**Figure 8:**
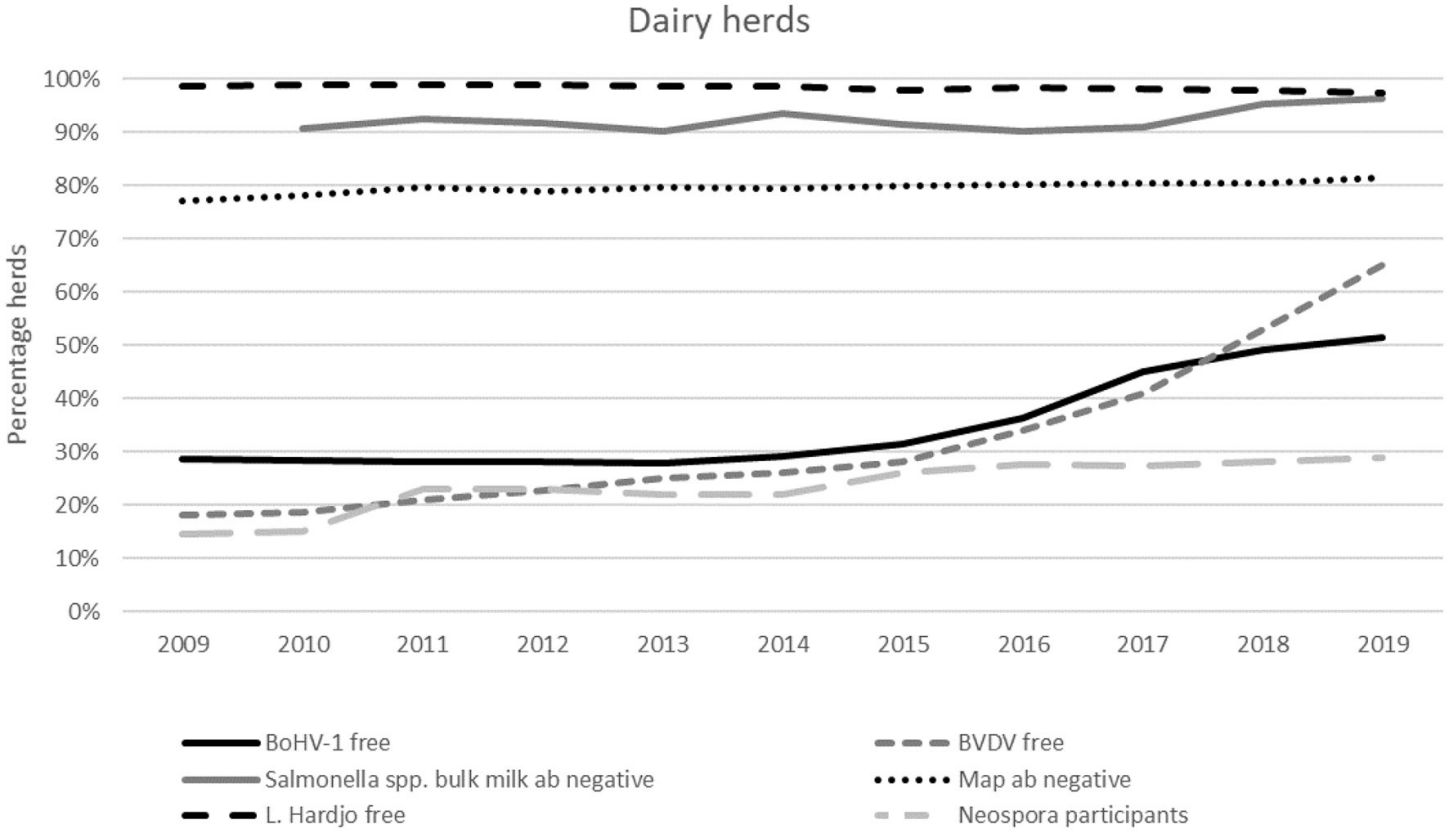
Dutch dairy herds with a free (BVDV, BoHV-1, *L*. Hardjo) or unsuspected (*Salmonella* spp., *Map*) status, or that participate in the *Neospora* monitoring program between 2009 and 2019.

For *Map*, ~80% of the Dutch dairy herds were assigned a preferred herd status (status A in MQAP or 6-10 in IPP). In the last decade, this proportion hardly changed. The other 20% of the herds had antibody positive cattle (status B or C) that have to be removed. From the start of the mandatory CP for *Salmonella* spp. until 2017, ~90% of the herds had bulk milk antibody negative results. Since 2018, additional actions have been taken to guide farmers where there is evidence of ongoing infection from bulk milk monitoring. This has been associated with an increased proportion of herds classified as unsuspected [on average 96.2% per measurement in 2019 (Figure 8)].

For BVDV and BoHV-1, the mandatory CPs have been in place since 2018 for dairy herds. From 2015 onwards, when initiatives were taken to develop national CPs for these two infections, voluntary participation rates and the proportion of herds classified as free already started to increase. Following the implementation of the national CP in dairy herds, the proportion of herds classified as BVDV free has increased further to almost 65% at the end of 2019. For the remaining 35% of herds, eight percent were classified as unsuspected, and the remaining 27% were in the process of achieving official free or unsuspected status. Some of these herds may not necessarily have had circulation of BVDV, given that it takes at least 10 months to obtain a free status. This period depends on the chosen route to become BVDV free and the BVDV infection status of the herd. At the end of 2019, the proportion of dairy herds with a BoHV-1 free status was 51%. A further 25% had an unsuspected status based on regular antibody-negative bulk milk tests. The remaining herds are vaccinated.

The monitoring program for *Neospora* does not aim to eliminate the infection from the herd but aims to monitor the status and provide insights into whether the herd is at risk for *Neospora* related abortion problems. The participation rate in this voluntary program showed a slight increase from 26% in 2015 to 29% of the Dutch dairy herds in 2019 (Figure 8).

#### Participation Rates in Non-dairy Herds

Non-dairy herds can participate voluntarily in five CP (there is no CP for *Neospora* available for non-dairy herds), but the CP participation rates for *Salmonella* spp. and *Map* are below five percent. In the other three CPs, for BVDV, BoHV-1 and *L*. Hardjo, the highest participation rates in non-dairy herds were observed in suckler herds and exceeded 10% in this herd type in 2019. The participation rates in young stock rearing farms are associated with the participation of dairy herds given that the two sectors were linked to each other. Participation rates in CPs by other herd types were negligible.

Most suckler herds that participated in the CPs for BVDV, BoHV-1 or *L*. Hardjo were classified as free. Therefore, the proportion of herds with a free status was similar to the proportion of herds that participated in the CP, meaning that herds that were not classified as “free” generally did not participate in the CP and had an unknown infection status. Reasons for farmers not to participate were often unrelated to the infection status of the herd.

**Figure 9:**
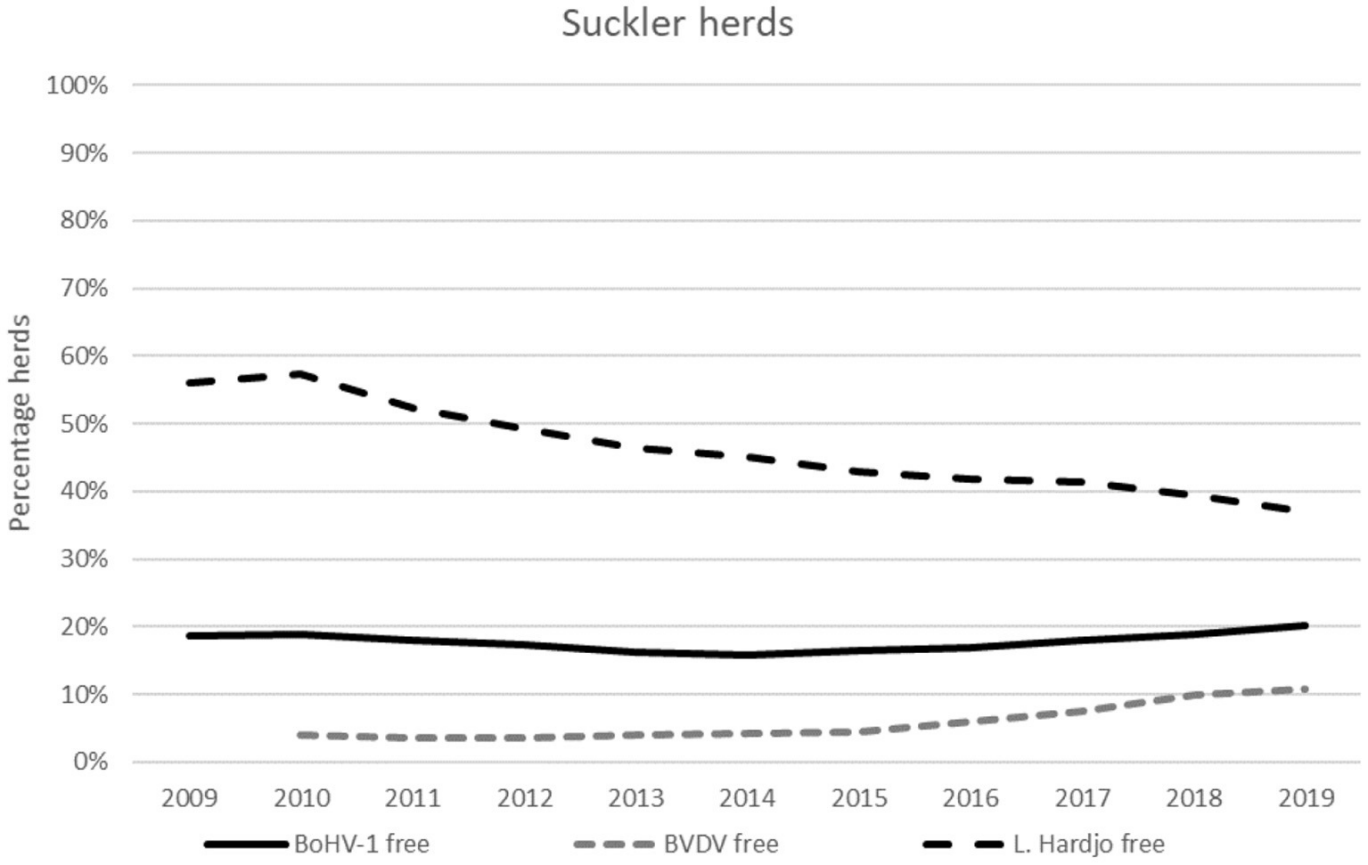
Dutch suckler herds that voluntarily participate in a CP for BoHV-1, BVDV or *L*. Hardjo and have a free status between 2009 and 2019.

For *L*. Hardjo, a steady decrease in the proportion of herds that participate and are subsequently classified as free was observed throughout the study period (Figure 9). This proportion decreased from 60% in 2009 to 37% in 2019. For both BVDV and BoHV-1, the proportion of participating free herds between 2009 and 2015 stayed the same. From 2015 to 2019 the proportion of herds participating in the CP for BVDV and BoHV-1 that were classified as free increased: from four to eleven percent for BVDV and from sixteen to twenty percent for BoHV-1.

### Change in Disease Prevalence Since the Implementation of Control Programmes

#### Decreasing Prevalence in Dairy Herds

**Figure 10:**
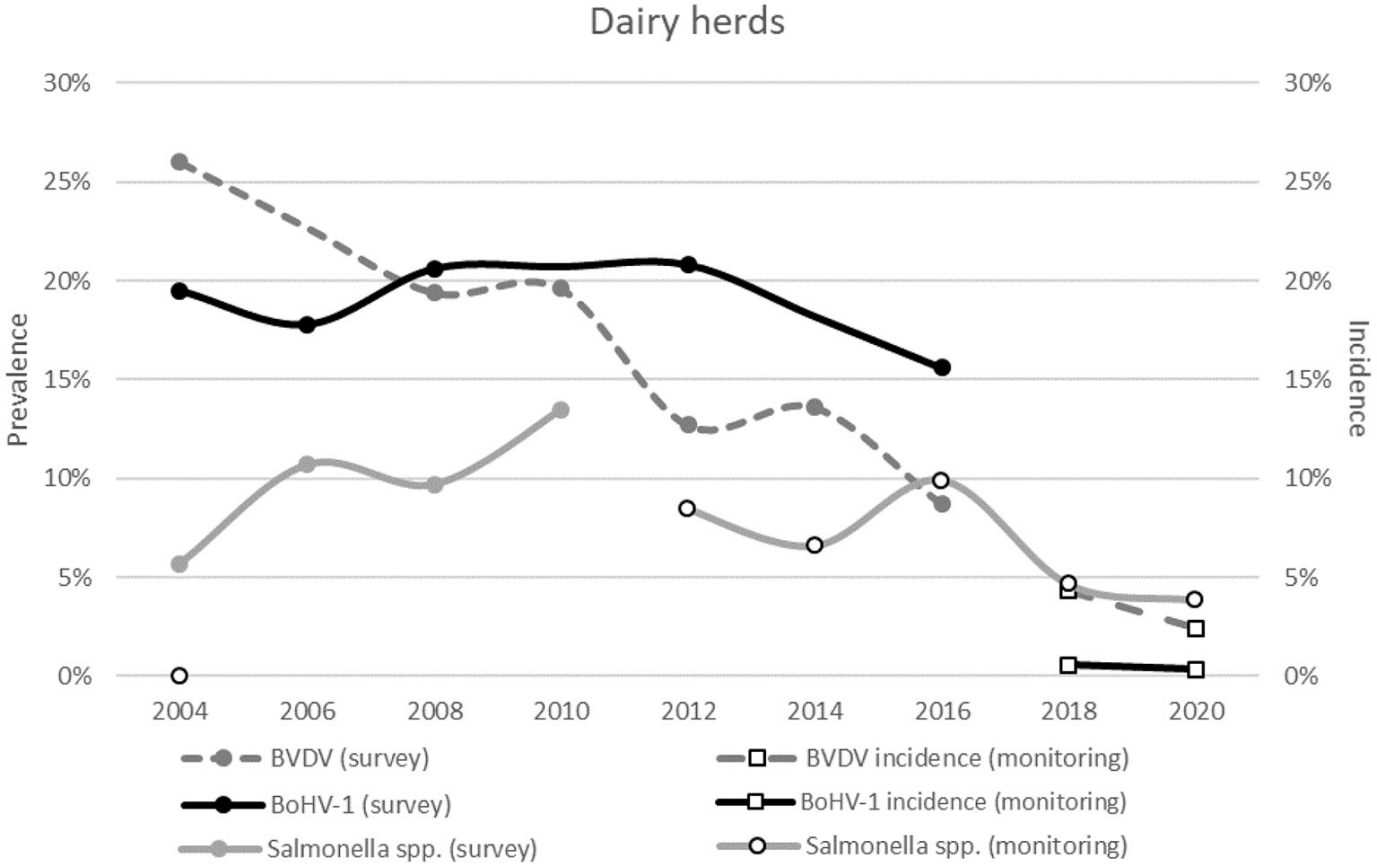
The prevalence in surveys conducted between 2004 and 2020 for BVDV, BoHV-1 and *Salmonella* spp. on Dutch dairy herds. The white markers with the black border indicate the incidence or prevalence based on the national monitoring program instead of a prevalence survey. The accompanying confidence intervals are presented in Appendix 2.

For three out of six infectious diseases, regular prevalence surveys were available in the Netherlands. For *L*. Hardjo no survey was conducted given that almost all herds were classified as “free.” For *Salmonella* spp., the prevalence showed an increase between the first survey in 2004 and 2010 (Figure 10). The most recent survey was done in winter 2009–2010, just after the start of the national control program, and showed that 13.5% (95% CI: 9.6–18.2) of the Dutch dairy herds were antibody positive. Thereafter, the infection status of each herd was monitored in the program and provided continuous information of the *Salmonella* spp. herd prevalence on a national level and made the bi-annual survey for *Salmonella* spp. superfluous. From 2009 on, the average percentage of herds in which antibodies against *Salmonella* spp. were detected in the national CP are presented and indicate a decrease in *Salmonella* spp. prevalence since the start of the national CP (Figure 10).

For BVDV and BoHV-1, the national herd-level prevalence in dairy herds decreased over time with increasing participation rates (and increasing numbers of herds classified as free or unsuspected) in the voluntary control programs (Figure 10). At the first survey in 2004, 26% (95% CI: 19.9–32.4%) of the herds had an indication of BVDV circulation. This percentage declined to 8.7% (95% CI: 5.2–13.7%) in the most recent survey in 2016. The prevalence of BoHV-1 also decreased, which was, however, not as marked as BVDV. In 2004, 19.5% (95% CI: 14.2–25.7%) of the Dutch dairy herds tested BoHV-1 antibody positive. In the most recent survey in 2016, 15.6% (95% CI: 12.6–19.1%) of the Dutch dairy herds still had antibodies. Since the implementation of the national programs the status of each herd is known, limiting the merit of prevalence surveys for BVDV and BoHV-1. The incidence is one of the parameters that is routinely monitored, which was calculated for BVDV as 4.3% (95% CI: 4.0–4.7%) in 2018–2019 and 2.4% (95% CI: 2.2–2.7%) in 2019-2020 (Figure 10). Since the implementation of the national BoHV-1 CP, the incidence in herds with a free or unsuspected status has been very low with 0.6% (95% CI: 0.4–0.7%) in 2018–2019 and 0.4% (95% CI: 0.3–0.5%) in 2019–2020.

More detailed information on the survey results is presented in Appendix 2.

For *Neospora*, no prevalence surveys were conducted. However, there was information on the post mortem findings in aborted fetuses submitted for post-mortal examination and results of serological sampling in aborting cattle at Royal GD. More than 95% of these fetuses were submitted from dairy herds.

**Figure 11:**
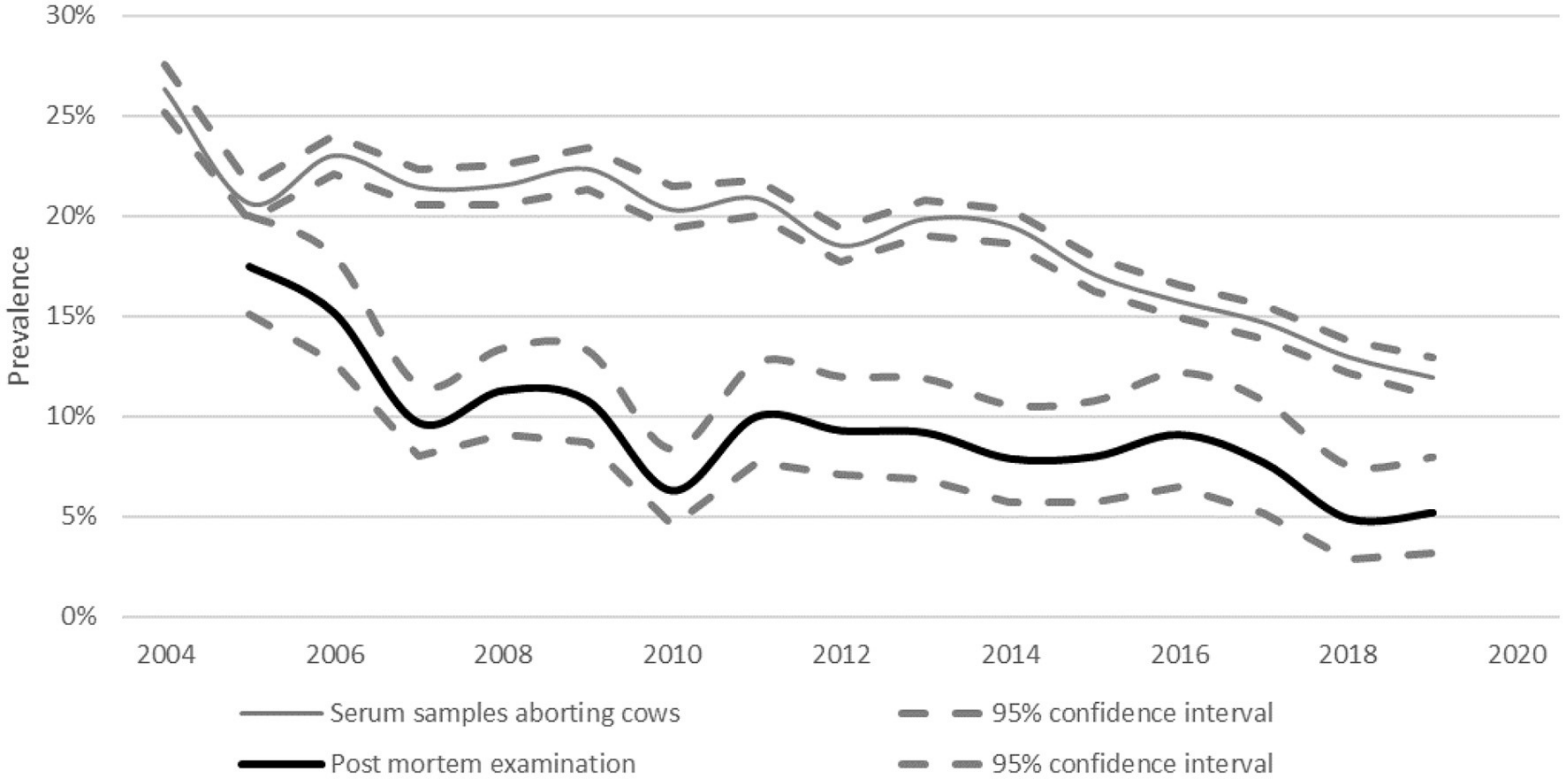
Percentage of abortions in which *Neospora* was diagnosed as the cause since 2004.

Since 2004 the proportion of fetuses in which *Neospora* was diagnosed as the cause of abortion has decreased from 17.5% (95% CI: 15.1–20.0%) in 2005 to 5.0% (95% CI: 3.2–8.0%) in 2019 (Figure 11). In the same period, the proportion of serological samples of aborting cows in which *Neospora* was diagnosed as probable cause of abortion, decreased significantly from 26.3% (95% CI: 25.2–27.5%) in 2004 to 11.9% (95% CI: 11.0–12.9%) in 2019 (Figure 11).

#### Decreasing Disease Prevalence's Over Time in Non-dairy Herds

**Figure 12:**
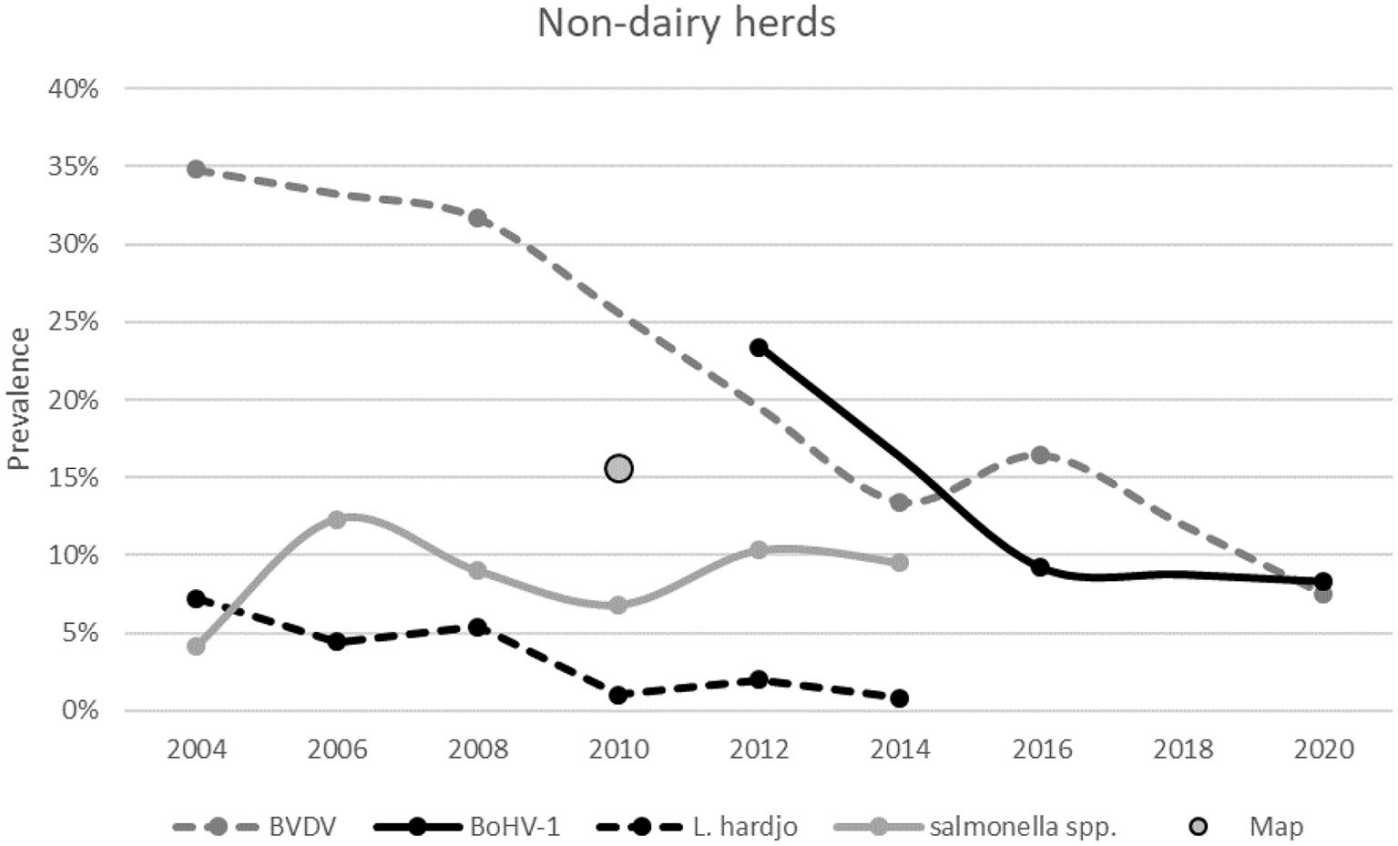
The herd prevalence from surveys conducted for BVDV, BoHV-1, *Salmonella* spp. and *Map* between 2004 and 2019 at Dutch non-dairy herds. The accompanying confidence intervals are presented in Appendix 2.

During the analyzed period, in non-dairy herds, a decrease in prevalence was observed for BVDV, BoHV-1 and *L*. Hardjo (Figure 12) while the proportion of herds classified as free, did not show a notable increase in the same period (Figure 9). The *L*. Hardjo prevalence decreased significantly from 7.2% (95% CI: 5.7–12.7%) in 2004 to 0.8% (95% CI: 0.2–2.2%) in 2014. During the studied period, the BVDV prevalence decreased from 34.8% (95% CI: 29.3–40.3%) in 2004 to 7.5% (95% CI: 4.3–11.9%) in 2020 and also the BoHV-1 prevalence decreased. During the first prevalence survey in 2012, 23.4% (95% CI: 16.4–31.2) of the non-dairy herds tested BoHV-1 positive. In 2020, this prevalence was significantly lower (8.3%, 95% CI: 4.9–12.9%).

For *Map*, only one survey was carried out, which indicated a herd prevalence of 15.6% (95% CI: 12.2–19.1%). For *Salmonella* spp., the prevalence in 2006 and 2014 was similar at ~10%.

More detailed information on the survey results is presented in Appendix 2.

### A High Health Status Is Associated With Lower Mortality

**Figure 13:**
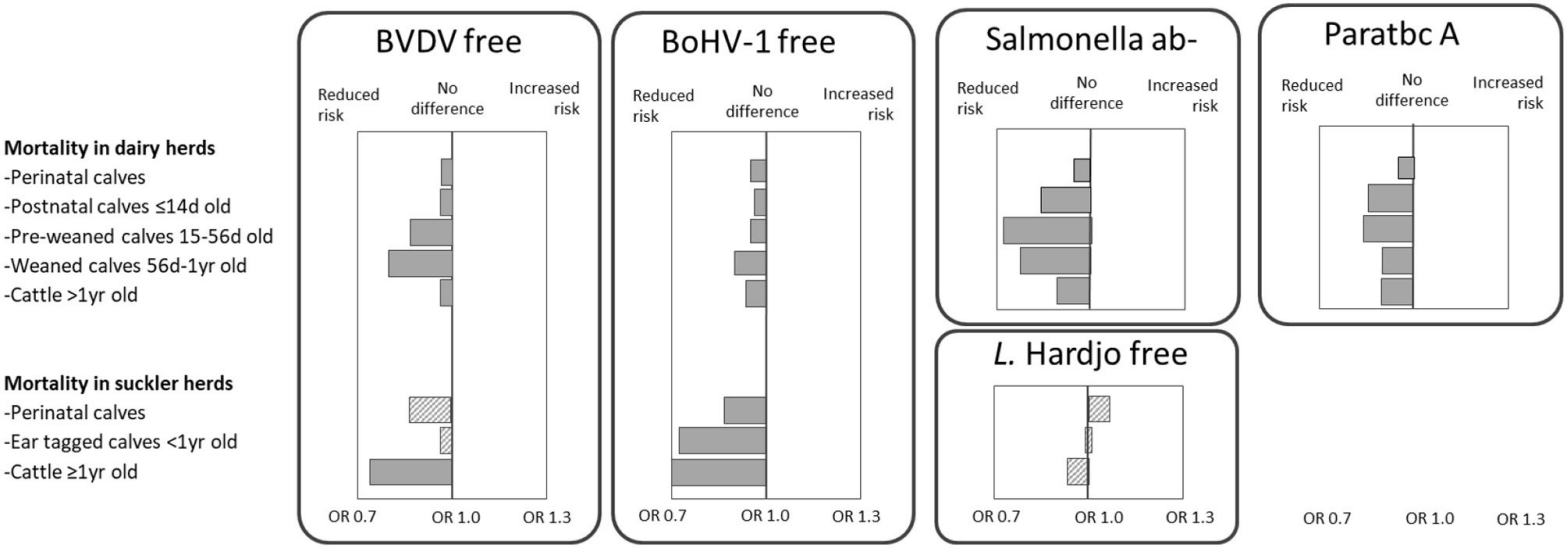
The association between a disease-free status/ antibody negative (ab–)/ status-A (ab–) and mortality in all dairy and all Dutch suckler cow herds in a multivariable population average logistic regression model between 2014 and 2019. Solid boxes represent significant deviations from the Dutch average, and dashed boxes represent non-significant results. Larger boxes represent more extreme odds ratios.

Dutch dairy herds with a favorable herd health status for BVDV, BoHV-1, *Salmonella* spp. or *Map* had significantly lower mortality rates compared to herds without a free, antibody negative or A status (Figure 13). The strongest protective associations between herd health and mortality were seen in the groups of pre-weaned and weaned calves.

In suckler herds, a BoHV-1 free status was associated with a significant lower mortality in all evaluated age groups of cattle (Figure 13). For BVDV, a protective effect of having a free status was also observed, although not significant in calves. For *L*. Hardjo no difference in mortality was found between *L*. Hardjo free herds relative to non-free herds. The odds ratios with the respective 95% confidence intervals are provided in Appendix 3.

## Discussion

This study described the control efforts for six endemic cattle infections between 2009 and 2019 in the Netherlands.

### The Dutch Approach in Disease Control

In the Netherlands, the six infections are at very different stages of control, ranging from only voluntary participation (*Neospora*), mandatory participation for dairy herds (BVDV, BoHV-1), or obligation to have a free (*L*. Hardjo) or unsuspected status for dairy herds (*Salmonella* spp., *Map*). Each of the CPs originally started voluntarily for both non-dairy and dairy herds. Participation rates were always highest for dairy herds, although these never exceeded 50% in the voluntary stages of the CPs before incentives to participate were implemented by the dairy processors. Initiatives were taken by the dairy processing industry to control and eliminate cattle infections from individual dairy herds through mandatory participation in CPs for BVDV, BoHV-1, *L*. Hardjo, *Salmonella* spp. and *Map*. For non-dairy herds, participation in all six CPs has remained voluntary. The main drivers for the dairy processing industry to control infections are not only to reduce the disease prevalence and disease associated losses but also to prevent the occurrence of zoonotic infections (i.e., salmonellosis or leptospirosis) and to deliver high quality products derived from healthy cows. Having high health statuses is important for the license to produce and the image of the sector and lead to increased consumer confidence in the products. Some export markets even demand products to originate from healthy cows.

The rules in each of the CPs are set to control and eliminate the specific infection from individual herds while taking factors as cost-effectiveness and minimal efforts for the farmer into account. In the Netherlands, this often results in CPs with multiple routes to achieve a free or unsuspected status. When possible, bulk milk surveillance is applied for dairy herds as a very cost- and labor-effective method to monitor the infection status of the herd. The sensitivity of bulk milk testing is often lower than the sensitivity of individual testing (37), which can be compensated by increasing the frequency of testing and the number of negative bulk milk tests required, which subsequently results in a higher herd sensitivity and earlier detection (72, 73). For non-dairy herds, individual sampling is the only option to control and eliminate infection and monitor the subsequent status. This whole herd sampling is labor-intensive and expensive. However, individual sampling for surveillance purposes can sometimes be done at slaughter to make it more efficient. The specific test characteristics of the tests used in the CPs are not discussed in this study. We acknowledge that test characteristics are very fundamental, but in our CPs, the test characteristics are incorporated in the design of the CPs. For tests with low specificity, confirmation tests are available, and low sensitivity is compensated by an increased sample size and/or test frequency. To increase the sensitivity of early detection of new infections, in all CPs, risk-based testing of high-risk animals is included, for example, by automatically testing serum samples of aborting cattle that are submitted for the mandatory brucellosis surveillance CP and testing of newly introduced cattle (while suspending the high health status until the result is known).

When a CP for an infection is developed and a testing strategy is chosen, the context situation, disease characteristics e.g., the routes of transmission, the prevalence, etc. and test characteristics i.e., sensitivity and specificity are taken into account. Over time, the implementation of a CP will lead to a reduction in within-herd and between-herd prevalence. When the prevalence reduces this can have an effect on the sensitivity and the specificity, which may result in a decreased validity of the original assumptions that were used when originally designing the CP. Although we acknowledge this factor, in general, the testing strategy in our CPs do not change over time given that changing the testing strategy may result in a lack of trust in the CP by farmers and their veterinarians. Additionally, when a new infection is introduced in a complete susceptible herd, we are confident that the within-herd prevalence will increase sufficiently to be detected by the testing strategies chosen in the current CPs.

The Dutch strategy in which participation in CPs starts voluntarily and becomes mandatory overtime, enables a review and revision of the CP to optimize the CP during the transition phase. Additionally, it provides the opportunity for farmers to start controlling the infection in their herds at their own preferred pace and thus helps to prepare farmers' mindset toward national control of the specific infection. A voluntary period before implementing a mandatory CP has the advantage that some herds are already free at the start of the mandatory CP. This makes it easier to control the risk of neighborhood contacts and purchase, given that it is possible to purchase cattle from herds with a similar or higher health status. Changes in the structure of CPs are, amongst others, initiated when certain aspects of the CP can be improved without hampering the efficiency of the CP (reducing labor or costs) or when the prevalence and incidence of the infection indicates a need for stricter regulations.

### Risks for (Re-)Introduction of Controlled Diseases

For *L*. Hardjo, the dairy processing industry is close to freedom from infection, and the prevalence in non-dairy herds is low as well. However, each year, several re-introductions occur, mainly through the import of cattle from countries with higher prevalence (74). For the other four infections i.e., BVDV, BoHV-1, *Salmonella* spp., and *Map*, (re-)introduction in herds with a free or unsuspected status occur, mainly because of introduction of infected cattle from cattle herds in the Netherlands. The introduction of cattle is a very important risk factor for disease introduction (21), and thus, the risk of purchase is controlled by requiring post-movement testing of introduced cattle originating from herds with a lower herd status in all CPs. Although these post-movement testing reduces the risk of undetected introduction of infections associated with the introduction of cattle into a herd, the test obligation does not entirely prevent the (re-)introduction of disease given that purchased animals have already been added to the herd before the infection status of the introduced animal is evaluated. Ideally, animals should be pre-screened before introducing them to the herd and/or quarantined until a post-movement negative test result is available. However, we are not allowed to set demands on the disease status of the traded cattle originating from herds that do not participate in the CP for these endemic diseases (i.e., the non-dairy herds). Additionally, quarantine is hardly ever done in dairy herds, and a notification from the CCP is often needed before the farmer submits the required samples. Therefore, in the CPs the “free” status is automatically suspended after purchase of cattle until it is proven that the purchased animal does not pose a risk (i.e., has an antibody or virus negative result) for the disease under control.

### Farmers Attitude Toward Disease Control

Many Dutch farmers aimed to eliminate infections in their herds when disease control was still in the voluntary stages. These farmers were keen on a high health status, wanted to avoid disease-related losses (sometimes based on earlier experience of losses), or perceived a high risk of the disease (either due to severity of signs or a high probability of introduction). These reasons are not unique for Dutch farmers as similar results were found in Great Britain (75). Other farmers only started to take measures when the costs of disease control were paid for by sectoral or public funds or when they were rewarded for having a high health status. The third group of farmers only started when they were obliged to control the disease by governmental or sectoral regulations. These differences in attitude to the control of diseases are in accordance with previous findings in the Netherlands on the mindset of farmers related to calf mortality (76). In the Netherlands, farmers' mindset related to mandatory disease control at national level is also influenced by a historic failure to eradicate BoHV-1. In 1998, a national CP that included vaccination was initiated with the aim to eradicate BoHV-1, after simulation studies showed that compulsory vaccination was needed to eradicate the virus (77). On 23 February 1999, the vaccination campaign was temporally postponed given that a batch of the vaccine was contaminated with BVDV type 2 (78). As a result, many farmers attributed clinical signs in their cattle to the vaccine, even though studies showed that not all reported signs could be blamed on vaccination (79, 80). Eventually, in December 2000, the mandatory control of BoHV-1 was suspended and it took another 18 years before a new attempt was made to control BoHV-1 on a national level.

### Monitoring Prevalence of Infections

The results of the biennial prevalence surveys show a decrease in prevalence over time for the infections under control. The success rate i.e., reduction in prevalence after implementing obligatory participation in the CPs, varies between infections and is multifactorial, depending among others on the differences in disease characteristics, the specific rules in the CP, and the demands set by the industry (participation vs. eradication).

The prevalence surveys indicated that when disease control measures were implemented in dairy herds the prevalence for these endemic infections also significantly decreased in non-dairy herds, even though participation rates remained low for non-dairy herds. Suckler herds may play a relevant role in the transmission of the six infections between herds in the Netherlands given that (i) these cattle are kept outside, (ii) suckler herds often trade and ii) calves are born regularly. In this herd type however, we observed reduced prevalences over time for infections that were under mandatory control in dairy herds. We therefore believe that disease control in one part of the population can also benefit the disease prevalence in the population that does not participate. Nevertheless, we cannot conclude that the reduction in prevalence can entirely be attributed to the performance of the CPs. Awareness of the disease may also have resulted in improved disease control in herds without official participation in a CP. Additionally, other changes in the cattle industry may have affected disease prevalence as well. In 2015, the milk quota was abolished. Subsequently, herd size increased untill governmental phosphate regulations were put into place that resulted in herd sizes reducing back to the level of July 2015. Generally, herd size was reduced by removing cattle that performed suboptimal. This may also have resulted in a decreased prevalence of several infections over time.

Given that participation in CPs is still voluntary for non-dairy herds, complete eradication from infection will probably not be reached. The prevalence of infection can become very low, but re-introduction through introduction of cattle will always be a risk. Additionally, given that eradication at national level is almost impossible without a national obligation to eliminate diseases, it will be very complicated to set demands on the disease status, when importing cattle, due to international trade regulations. However, eradication may not be necessary to achieve the goals of stakeholders, such as safeguarding future access to international dairy markets.

### Association Between Participation in a Disease Control Program and Mortality

The implementation of CPs for specific infections improves animal health and welfare, and reduce disease-related costs and labor involved in the treatment of diseased animals. In this study, we showed that herds with a free or unsuspected status for the evaluated infections had lower mortality rates in calves and cows than herds with an unknown status. Infectious diseases are risk factors for mortality and culling as previously reported for BVD (81, 82), salmonellosis (83, 84), and paratuberculosis (85–88). Nevertheless, the effect estimates presented in this study are likely to be underestimated, given that some of the herds with an unknown status will also be free from infection. On the other hand, management practices and biosecurity measures in herds with a disease-free or unsuspected status may differ from those in herds with unknown infection statuses. These management practices might also be associated with reduced mortality. Data on farmers' management were unavailable and were not included in this study.

### Comparability of Control Programs for Cattle Diseases Between Countries

CPs to control and eradicate infections are to be supported, differences in herd health status within and between countries pose a risk when trading cattle from areas with a higher disease prevalence to areas with a lower prevalence (5). For cattle diseases with no or only limited regulation at EU level, the design of a CP is often tailored to the country-specific situation resulting in considerable heterogeneity in CP design between countries (89). The tailored CPs are designed cost-effectively while taking factors such as prevalence, incidence, and risks into account. These CPs are often a better fit for each situations' specific needs than the input-based CPs in EU regulations. However, the heterogeneity of CPs complicates comparison of the free statuses between regions and countries. In an assessment in which 32 European countries participated, it appeared that there are 24 different bovine infections for which CPs exist in at least one European country while there is no or limited regulation on EU level (90). In the Netherlands, a CP is in place for 11 out of the 24 cattle diseases. For the other 13 cattle infections that were evaluated no CP is available yet for varying reasons. Some of these infections are not so relevant given that they do not occur in the Netherlands e.g., Epizootic haemorrhagic disease, Surra and Lumpy skin disease. Other infections are not yet under control but may be in the future. These infections were however not prioritized over the 11 controlled endemic infections. The Netherlands is free of five out of the 11 diseases for which a CP is in place, i.e., enzootic bovine leukosis, bluetongue, anthrax, trichomonosis, and bovine genital campylobacteriosis. The other six controlled cattle diseases are either endemic (BVD, IBR, salmonellosis, and paratuberculosis) or occur sporadically (leptospirosis) and were included in this evaluation. Based on the detailed description of the specific diseases and the CP in place, defined input parameters can be included in an output-based framework. For BVDV, this has already been done (91, 92). For other diseases, the information presented in this study, can serve as a basis to expand output based frameworks that are developed for cattle diseases. Additionally, this manuscript presents how cattle diseases with no or limited regulation on EU level are controlled in the Netherlands, a gross exporting country with high cattle density, a high rate of cattle movements between herds and a very high-quality data infrastructure. The description of the CP design may provide guidance to other countries that want to start the control of these cattle diseases.

## Data Availability Statement

The data analyzed in this study is subject to the following licenses/restrictions: Confidential data. Requests to access these datasets should be directed to i.santman@gdanimalhealth.com.

## Author Contributions

IS-B was the primary author of the manuscript. MM added knowledge on the testing process, helped in writing the paper, and proof read the paper. MW provided input on *Salmonella* spp. and *Map*. LD provided input on BVDV. HW provided input on BoHV-1. KB provided input on *L*. Hardjo. TD provided input on *Neospora* and *Map*. AR is the CP manager and proofread the relevant parts of the paper. All experts assisted in the writing of the parts of the paper about the CPs concerning the infection that met their expertise. JH, SS, MM, MW, MB, AV, and GS edited draft versions of the paper and provided advice for improvement. MB and AV provided veterinary epidemiologic expertise and conducted the analysis on the prevalence estimations over time. GS acted as supervisor of the group and provided scientific and epidemiologic input. All authors participated in the writing of this manuscript, have proofread the final version of the manuscript and agree with its contents.

## Conflict of Interest

IS-B, MM, MW, LD, HW, KB, TD, AR, AV, and GS are employed by Royal GD, which runs most of the CPs described in this paper. All authors declare that the research was conducted in the absence of any commercial or financial relationships that could be construed as a potential conflict of interest.

## Publisher's Note

All claims expressed in this article are solely those of the authors and do not necessarily represent those of their affiliated organizations, or those of the publisher, the editors and the reviewers. Any product that may be evaluated in this article, or claim that may be made by its manufacturer, is not guaranteed or endorsed by the publisher.
