# Corrigendum: Control and Eradication Programs for Six Cattle Diseases in the Netherlands

**DOI:** 10.3389/fvets.2021.817576

**Published:** 2021-12-08

**Authors:** I. M. G. A. Santman-Berends, M. H. Mars, M. F. Weber, L. van Duijn, H. W. F. Waldeck, M. M. Biesheuvel, K. M. J. A. van den Brink, T. Dijkstra, J. J. Hodnik, S. A. J. Strain, A. de Roo, A. M. B. Veldhuis, G. van Schaik

**Affiliations:** ^1^Department of Research and Development, Royal GD, Deventer, Netherlands; ^2^Department of Population Health Sciences, Faculty of Veterinary Medicine, Utrecht University, Utrecht, Netherlands; ^3^Department of Cattle Health, Royal GD, Deventer, Netherlands; ^4^Department of Production Animal Health, Faculty of Veterinary Medicine, University of Calgary, Calgary, AB, Canada; ^5^Veterinary Faculty, University of Ljubljana, Ljubljana, Slovenia; ^6^Animal Health and Welfare Northern Ireland, Dungannon, United Kingdom

**Keywords:** disease control, sound control, endemic diseases, control programs, monitoring, surveillance, dairy, beef

In the original article, there was an error in the **Title** and **Abstract, Paragraph One**. We used the phrase “non-regulated” for cattle diseases that are in fact listed in the New Animal Health Law that went into force in 2021. The corrected title and paragraph appear below.

**Title**

**Control and Eradication Programs for Six Cattle Diseases in the Netherlands**

**Abstract**

Within the European Union, infectious cattle diseases are categorized in the Animal Health Law. No strict EU regulations exist for control, evidence of disease freedom, and surveillance of diseases listed other than categories A and B. Consequently, EU member states follow their own varying strategies for disease control. The aim of this study was to provide an overview of the control and eradication programs (CPs) for six cattle diseases in the Netherlands between 2009 and 2019 and to highlight characteristics specific to the Dutch situation. All of these diseases were listed as C,D or E in the New Animal Health Law.

The authors apologize for these errors and state that this does not change the scientific conclusions of the article in any way. The original article has been updated.

## Publisher's Note

All claims expressed in this article are solely those of the authors and do not necessarily represent those of their affiliated organizations, or those of the publisher, the editors and the reviewers. Any product that may be evaluated in this article, or claim that may be made by its manufacturer, is not guaranteed or endorsed by the publisher.

