# Supplementary material for: Control and Eradication Programs for Six Cattle Diseases in the Netherlands
Source: Front Vet Sci. 2021 Aug 18;8:670419. doi: 10.3389/fvets.2021.670419 (PMC8418201; doi:10.3389/fvets.2021.670419)
Supplement: Supplementary file 1 [file Data_Sheet_1.docx]

**Appendix 1. References to detailed regulations of the disease control programs in place for cattle in the Netherlands (in Dutch), accessed on December 2020**

**BVDV**

- <https://www.ibrbvd.nl/wp-content/uploads/2018/04/BVD-tbv-geborgde-dierenarts.pdf>
- Official regulation screening, removing PI’s and monitoring in young stock route: <https://www.zuivelnl.org/uploads/images/Diergezondheid-dierenwelzijn/Protocol-ZuivelNL-statusbeheer-BVD-route-intake-virus-bewaking-jongvee-antistoffen-versie-1.4.pdf>
- Official regulation bulk milk monitoring route: <https://www.zuivelnl.org/uploads/images/Diergezondheid-dierenwelzijn/Protocol-ZuivelNL-statusbeheer-BVD-route-tankmelk-versie-1.4.pdf>
- Official regulation monitoring young stock antibodies route: <https://www.zuivelnl.org/uploads/images/Diergezondheid-dierenwelzijn/Protocol-ZuivelNL-statusbeheer-BVD-route-jongvee-antistoffen-versie-1.4.pdf>
- Official regulation ear notch testing route: <https://www.zuivelnl.org/uploads/images/Diergezondheid-dierenwelzijn/Protocol-ZuivelNL-statusbeheer-BVD-route-oorbiopten-versie-1.3.pdf>

**BoHV-1**

- <http://www.ibrbvd.nl/wp-content/uploads/2016/07/IBR-tbv-geborgde-dierenarts.pdf>
- Official regulation screening, removing positive animals and monitoring in bulk milk route: <https://www.zuivelnl.org/uploads/images/Diergezondheid-dierenwelzijn/Protocol-ZuivelNL-statusbeheer-IBR-vrij-route-intake-bloed-bewaking-tankmelk-versie-1.1.pdf>
- Official regulation bulk milk screening route: <https://www.zuivelnl.org/uploads/images/Diergezondheid-dierenwelzijn/Protocol-ZuivelNL-statusbeheer-IBR-vrij-route-tankmelk-versie-1.1.pdf>
- Official regulation vaccination route:
  https://www.zuivelnl.org/uploads/images/Diergezondheid-dierenwelzijn/Protocol-ZuivelNL-statusbeheer-IBR-vrij-route-vaccinatie-versie-1.2.pdf

***L*. Hardjo**

- <https://www.gddiergezondheid.nl/~/media/Files/Regelementen/reglementen%20rund/Reglement%20Certificering%20Leptospira%20hardjo%20bij%20runderen%202002%20pdf.ashx>
- <https://www.gddiergezondheid.nl/producten%20en%20diensten/producten/rundvee/leptospirose-aanpak/leptospirose-vrij%20certificering%20melkvee>
- <https://www.gddiergezondheid.nl/diergezondheid/dierziekten/leptospirose-rund>

***Salmonella* spp.**

- Unsuspected CP, Royal GD:
  - Dairy herds: <https://www.gddiergezondheid.nl/~/media/Files/Regelementen/reglementen%20rund/Reglement%20GD%20Programma%20Salmonella%20Onverdacht%20voor%20melkveebedrijven.ashx>
  - Non-dairy herds: https://www.gddiergezondheid.nl/~/media/Files/Regelementen/reglementen%20rund/Reglement%20Salmonella%20Jongveemonitor.ashx
- Mandatory CP for dairy herds, NZO: https://www.nzo.nl/documenten/, ‘witte lijst salmonella’

***Map***

- Milk quality assurance programme, Royal GD; https://www.gddiergezondheid.nl/~/media/Files/Regelementen/reglementen%20rund/Regelement%20Paratubercolose%20Programma.ashx
- Intensive paratuberculosis programme: https://www.gddiergezondheid.nl/~/media/Files/Regelementen/reglementen%20rund/Reglement%20Intensief%20Programma%20Paratuberculose%20bij%20Runderen%202005.ashx

***Neospora***

- https://www.gddiergezondheid.nl/diergezondheid/dierziekten/neospora-rund

**Appendix 2. Survey results for endemic cattle infections in the Netherlands from 2004 until 2020**

Table 1. National survey results in dairy herds in the Netherlands

|  | Herd level prevalence (95% CI) | | | | Herd level incidence (95% CI) | |
| --- | --- | --- | --- | --- | --- | --- |
| Year | BoHV-1 | BVDV | *Salmonella* spp.* | *Neospora* diagnosis in aborted fetuses | BoHV-1 | BVDV |
| 2004 | 19.5%  (14.2-25.7%) | 26.0%  (19.9-32.4%) | 5.7%  (2.5-11.0%) | 17.5%  (15.1-20.0%) |  |  |
| 2006 | 17.8%  (13.5-22.8%) |  | 10.7%  (6.6-16.1%) | 15.2%  (12.7-18.0%) |  |  |
| 2008 | 20.6%  (16.6-25.0%) | 19.4%  (15.6-23.7%) | 9.7%  (6.4-13.9%) | 11.3%  (9.1-13.4%) |  |  |
| 2010 | 20.7%  (16.8-25.1%) | 19.6%  (15.2-24.6%) | 13.5%  (9.6-18.2%) | 6.3%  (4.6-8.4%) |  |  |
| 2012 | 20.8%  (16.9-25.2%) | 12.7%  (9.2-17.3%) | 8.5%  (8.0-8.9%) | 9.3%  (7.1-12.0%) |  |  |
| 2014 | 18.2%  (14.7-22.2%) | 13.6%  (9.3-18.9%) | 6.6%  (6.2-7.0%) | 7.9%  (5.7-10.6%) |  |  |
| 2016 | 15.6%  (12.5-19.1%) | 8.7%  (5.2-13.6%) | 9.9%  (9.4-10.4%) | 9.1%  (6.5-12.2%) |  |  |
| 2018 |  |  | 4.7%  (4.0-5.0%) | 4.9%  (2.9-7.6%) | 0.6%  (0.4-0.7%) | 4.3%  (4.0-4.7%) |
| 2020 |  |  | 3.8%  (3.5-4.2%) | 5.2%  (3.2-8.0%) | 0.4%  (0.3-0.5%) | 2.4%  (2.0-2.7%) |

*Before 2012 the results that are shown originate from the bi-annual survey. From 2012 on the results that are shown originate from the national CP

Table 2. Results of screening for *neospora* in dairy herds in the Netherlands

|  | Animal level prevalence (95% CI) | |
| --- | --- | --- |
| Year | Serological sampling of aborting cattle | aborted fetuses |
| 2004 | 26.3% (25.2-27.5%) |  |
| 2005 | 20.6% (19.8-21.6%) | 17.5% (15.1-20.0%) |
| 2006 | 23.0% (22.1-24.0%) | 15.2% (12.7-18.0%) |
| 2007 | 21.5% (20.6-22.3%) | 9.7% (8.0-11.6%) |
| 2008 | 21.5% (20.6-22.6%) | 11.3% (9.1-13.4%) |
| 2009 | 22.4% (21.4-23.4%) | 10.8% (8.7-13.3%) |
| 2010 | 20.3% (19.4-21.5%) | 6.3% (4.6-8.4%) |
| 2011 | 20.9% (17.7-19.4%) | 10.0% (7.7-12.7%) |
| 2012 | 18.5% (17.7-19.4%) | 9.3% (7.1-12.0%) |
| 2013 | 19.9% (19.0-20.8%) | 9.2% (6.9-11.9%) |
| 2014 | 19.5% (18.7-20.4%) | 7.9% (5.7-10.6%) |
| 2015 | 17.1% (16.2-20.4%) | 8.0% (5.7-10.8%) |
| 2016 | 15.7% (15.0-16.6%) | 9.1% (6.5-12.2%) |
| 2017 | 14.9% (13.9-15.5%) | 7.7% (5.2-10.8% |
| 2018 | 13.0% (12.1-13.8%) | 4.9% (2.9-7.6%) |
| 2019 | 11.9% (11.0-12.9%) | 5.2% (3.2-8.0%) |

Table 3. National survey results in non-dairy herds in the Netherlands

|  | Herd level prevalence (95% CI) | | | |  |
| --- | --- | --- | --- | --- | --- |
| Year | BoHV-1 | BVDV | *Salmonella* spp. | *L*. Hardjo | *Map* |
| 2004 |  | 34.8%  (29.3-40.3%) | 4.1%  (1.9-7.6%) | 7.2%  (1.5-19.9%) |  |
| 2006 |  |  | 12.3%  (7.7-18.4%) | 4.4%  (2.1-6.8%) |  |
| 2008 |  | 31.7%  (25.9-37.9%) | 9.0%  (4.3-13.7%) | 5.4%  (2.4-11.7%) |  |
| 2010 |  |  | 6.8%  (2.2-11.4%) | 1.0%  (0.02-4.9%) | 15.6%  (12.2-19.1%) |
| 2012 | 23.4%  (16.4-31.2%) |  | 10.3%  (5.8-17.7%) | 2.0%  (0.04-4.9%) |  |
| 2014 |  | 13.4%  (7.3-21.8%) | 9.5%  (5.9-21.1%) | 0.8%  (0.2-2.2%) |  |
| 2016 | 9.2%  (5.1-14.9%) | 16.4%  (12.5-21.1%) |  |  |  |
| 2018 |  |  |  |  |  |
| 2020 | 8.3%  (4.9-12.9%) | 7.5%  (4.3-11.9%) |  |  |  |

**Appendix 3. Results of a multivariable population-averaged Poisson regression models in all Dutch dairy and suckler herds where the association between mortality and the herd status are studied**

|  |  |  | Mortality^2^ | | | | |
| --- | --- | --- | --- | --- | --- | --- | --- |
|  | Infection |  | Perinatal calves | Postnatal calves <14d | Pre-weaned calves 15-56d | weaned calves 56d-1yr | Cattle ≥1yr |
| Dairy | BVDV | Unknown^1^ | Ref. | Ref. | Ref. | Ref. | Ref. |
|  |  | Free | 0.96  (0.95-0.97) | 0.96  (0.94-0.99) | 0.90  (0.89-0.93) | 0.85  (0.83-0.88) | 0.96  (0.95-0.98) |
|  | BoHV-1 | Unknown^1^ | Ref. | Ref. | Ref. | Ref. | Ref. |
|  |  | Free | 0.95  (0.93-0.96) | 0.96  (0.94-0.99) | 0.95  (0.92-0.98) | 0.92  (0.89-0.95) | 0.94  (0.92-0.95) |
|  | Sal^3^ | Bulk milk antibody positive | Ref. | Ref. | Ref. | Ref. | Ref. |
|  |  | Antibody negative | 0.94  (0.92-0.96) | 0.86  (0.83-0.89) | 0.75  (0.72-0.78) | 0.82  (0.79-0.85) | 0.91  (0.88-0.93) |
|  | *Map* | Status B/C | Ref. | Ref. | Ref. | Ref. | Ref. |
|  |  | Status A/ 6-10 | 0.95  (0.94-0.97) | 0.88  (0.86-0.91) | 0.87  (0.84-0.96) | 0.87  (0.84-0.96) | 0.91  (0.89-0.92) |
|  |  |  | Perinatal calves | Ear-tagged calves <1yr |  |  | Cattle ≥1yr old |
| Suckler | BVDV | Unknown^1^ | Ref. | Ref. |  |  | Ref. |
|  |  | Free | 0.89  (0.77-1.02)^ns^ | 0.95  (0.84-1.08)^ns^ |  |  | 0.80  (0.68-0.94) |
|  | BoHV-1 | Unknown^1^ | Ref. | Ref. |  |  | Ref. |
|  |  | Free | 0.88  (0.77-1.00) | 0.75  (0.66-0.85) |  |  | 0.70  (0.59-0.84) |
|  | *L.* Hardjo | Unknown^1^ | Ref. | Ref. |  |  | Ref. |
|  |  | Free | 1.07  (0.96-1.19)^ns^ | 1.00  (0.90-1.10)^ns^ |  |  | 0.93  (0.81-1.06)^ns^ |

Ns= not significantly different

^1^Unknown=participating in the CP but without the preferred status or not participating in the CP

^2^The results represent the incidence rate ratios together with the 95% confidence interval (95% CI) of having a ‘free’ status relative to all other herds. Besides the infection status, herd size, milk production, purchase, antimicrobial usage, location, replacement percentage, growth in herd size, season and a trend in time were also included as covariates in the model (results not presented).

^3^ Sal=*salmonella* spp. Note: results are shown for herds with positive or negative bulk milk test results in the quadrimester of the year. A single negative bulk milk test is neither equivalent to an unsuspected status (voluntary CP) nor to Level 1 (mandatory CP) although there is a high correlation between the bulk milk status and the CP status.
